# GSCs differentiation model-informed nanotherapy: dual-functional brain-targeting liposomes with iRGD modification for co-delivery of osimertinib and bortezomib to combat radioresistant glioblastoma

**DOI:** 10.1038/s41419-025-08083-0

**Published:** 2025-10-21

**Authors:** Cuiying Xie, Jieyi Wu, Han Yang, Ancheng Gu, Wanwen Cao, Xiangcao Yao, Zhiyong Li, Yuchun Niu, Jianlong Li, Zhongyuan Xu, Bohong Cen

**Affiliations:** 1 Clinical Pharmacy Center, Nanfang Hospital, Southern Medical University, Guangzhou, 510515 Guangdong China; 2 School of Pharmaceutical Sciences, Southern Medical University, Guangzhou, 510515 Guangdong China; 3 The Department of Plastic and Cosmetic Surgery, Nanfang Hospital, Southern Medical University, Guangzhou, 510515 Guangdong China; 4 Department of Neurosurgery, Nanfang Hospital, Southern Medical University, Guangzhou, 510515 Guangdong China; 5 The First People’s Hospital of Foshan, Cancer Hospital, Foshan, 528000 Guangdong China; 6 Department of Orthopedic Surgery, Nanfang Hospital, Southern Medical University, Guangzhou, 510515 Guangdong China

**Keywords:** CNS cancer, Cancer models, Cancer therapeutic resistance, Radiotherapy

## Abstract

Radiation resistance in glioblastoma (GBM) poses a persistent clinical hurdle, driven in part by hyperactivated EGFR and NF-κB signaling. To recapitulate post-radiation tumor recurrence, we engineered radioresistant glioblastoma stem cells (GSCs) from U87-derived GSCs via 13 cycles of 5Gy irradiation (IR), yielding differentiated radioresistant progeny cells (Diff) that mimic the aggressive phenotype of recurrent GBM. Integrative analysis of RNA sequencing data from parental U87 cells, GSCs, and Diff cells—along with the TCGA database—identified coordinated EGFR and NF-κB (RelA/p65) signaling as central mediators of therapeutic resistance. Leveraging this insight, we designed iRGD-modified liposomes (iRGD-OB-LP) for targeted co-delivery of Osimertinib (EGFR inhibitor) and Bortezomib (NF-κB suppressor). These liposomes exhibited enhanced tumor penetration, sustained release kinetics, and dual pathway inhibition, which collectively prolonged radiation-induced DNA damage, attenuated cancer stemness, and amplified apoptotic cell death. In vivo, iRGD-OB-LP achieved tumor-specific biodistribution, synergized with radiotherapy to suppress tumor progression, and extended survival without systemic toxicity. By bridging a radioresistant GBM model with mechanism-driven nanotherapy, this work provides a translatable blueprint for dismantling therapeutic resistance in GBM through precision multi-targeting.

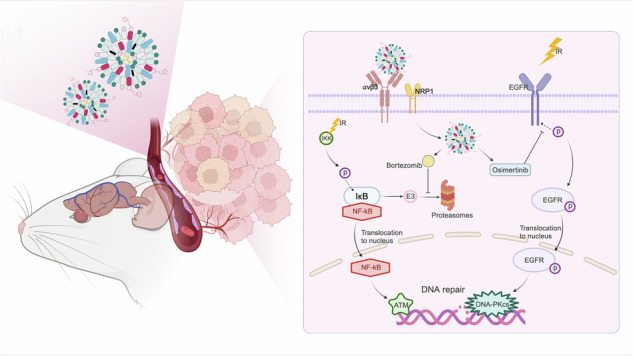

## Introduction

The therapeutic landscape of glioblastoma (GBM) remains among the most formidable challenges in neuro-oncology [1]. As a WHO grade IV primary brain malignancy, GBM exhibits aggressive proliferative patterns and inevitable recurrence [2] despite maximal treatment involving surgical resection, radiotherapy, and temozolomide chemotherapy [3]. The median survival stagnates at 14–16 months [4], a dismal prognosis exacerbated by therapeutic resistance mechanisms rooted in tumor heterogeneity and glioblastoma stem cells (GSCs) plasticity [5, 6]. Following conventional therapy, surviving GSCs evade apoptosis, rapidly reactivate differentiation programs, and repopulate treatment-refractory tumors [7–9]—a phenomenon recapitulated in our radioresistant GSCs differentiation model. This biological mimicry underscores the urgency to dismantle radioresistance pathways governing GSCs persistence and recurrence.

Radioresistance in GBM is orchestrated by compensatory survival signaling, particularly through nuclear factor-κB (NF-κB) and epidermal growth factor receptor (EGFR) cascades. NF-κB p65/RelA promotes radiation-induced DNA damage repair by enhancing homologous recombination (HR) via ATM kinase activation [10, 11], while nuclear EGFR collaborates with DNA-PKcs to drive nonhomologous end joining (NHEJ) [12, 13]. Clinically, hyperactivation of these pathways in recurrent GBM correlates with accelerated relapse and poor survival [14–16]. Pharmacological inhibition of NF-κB (via proteasome blockade, such as Bortezomib) and EGFR (via tyrosine kinase inhibition, such as Osimertinib) presents a rational dual-targeting strategy to cripple repair mechanisms and resensitize tumors to radiation [17–20]. However, traditional systemic administration of Bortezomib and Osimertinib faces critical barriers: (1) Dose-limiting toxicity from off-target proteasome inhibition; (2) Inadequate intracranial bioavailability due to blood–brain barrier (BBB) exclusion; (3) Failure to penetrate the dense glioblastoma parenchyma even at leaky blood–brain tumor barrier (BBTB) sites [21–23].

To address these challenges, we engineered an iRGD-modified liposomal platform for brain-targeted co-delivery of Osimertinib and Bortezomib. iRGD, a tumor-penetrating cyclic peptide, exploits αvβ3/αvβ5 integrin overexpression on GBM neovasculature for primary targeting, followed by neuropilin-1 (NRP1)-mediated tissue penetration [24–28]. When conjugated to nanoliposomes, this peptide not only breaches the BBB/BBTB but also facilitates deep tumor penetration via NRP1-dependent transcytosis—capabilities unattainable by free drugs or passive nanoparticle accumulation [29, 30]. The combinatorial payload strategically disrupts radioresistance: Bortezomib stabilizes IκB to sequester NF-κB in the cytoplasm, blocking p65-mediated HR repair [11, 18], while Osimertinib suppresses EGFR nuclear translocation and DNA-PKcs activation, impairing NHEJ [12, 13, 19, 20]. Mechanistic synergy between these agents may amplify radiosensitization while mitigating compensatory signaling.

Beyond conventional drug delivery paradigms, our therapeutic design integrates functional insights from a radioresistant GSCs differentiation model. By employing tumor cells derived from post-radiation GSCs, this system uniquely replicates the molecular evolution of clinical recurrence—an approach critical for validating pathway interdependencies and nanoparticle efficacy in pathophysiologically relevant microenvironments. Herein, we characterize the dual-loaded iRGD-liposomes for physicochemical stability, tumor-selective biodistribution, and radiosensitization potency across in vitro and orthotopic GBM models. This study pioneers a GSCs biology-informed nanotherapeutic strategy to dismantle key resistance axes, offering a blueprint for precision intervention in radioresistant glioblastoma.

## Materials and methods

### Preparation and characterization of liposomes

HSPC, CHO-HP, DSPE-mPEG2000, and DSPE-PEG2000-MAL (AVT, China) were dissolved in 10 mL of chloroform at a molar ratio of 52:47:0.1:1, respectively, and heated in a water bath at 60 °C to ensure complete dissolution. The mixture was vacuum-evaporated for 30 min using a rotary evaporator, forming a thin, uniform lipid film on the flask wall. The film was rehydrated by adding 20 mL of deoxygenated PBS buffer (250 mM (NH_4_)_2_SO_4_, pH adjusted to 4.0 with phosphoric acid) and rotary hydration for 30 min.

The iRGD peptide (Guoping Pharmaceutical Co., LTD, China) solution was then added, sealed, and the sample was agitated at 250 rpm at room temperature for 12 h. Following this, the liposomes were sonicated at 60 °C for 2 h and passed through a 100 µm polycarbonate membrane using an extruder, a process repeated six to eight times to achieve uniformity.

The liposomes were transferred to a 10 kDa molecular weight cutoff dialysis bag (Servicebio, China) and dialyzed in 1 L of deionized water containing 0.9% NaCl (pH adjusted to 7.4 with NaOH) for 1 h at room temperature. This dialysis step was repeated with fresh dialysate to establish a pH gradient. Osimertinib (2 mg, Selleck, China) and Bortezomib (2 mg, Selleck, China), dissolved in 1 mL of anhydrous ethanol, were then added to the liposome samples. The mixture was shaken at 250 rpm at 60 °C for 1 h to encapsulate the drugs.

Finally, the liposomes were placed in a 10 kDa cutoff dialysis bag and dialyzed again using the same method to remove free iRGD and unencapsulated drugs. The final iRGD-drug-loaded liposomes were collected. The size, polydispersity index (PDI), and zeta potential of the samples were characterized using a Zetasizer Nano ZS.

### Cell culture and construction of cell models

U87MG (U87) cell line (human malignant glioblastoma multiforme cell line, ATCC Number: HTB-14) was purchased from the Cell Bank of Type Culture Collection of the Chinese Academy of Sciences (Shanghai, China). Glioblastoma stem cells (U87GSCs, GSCs) were cultured in serum-free neural stem cell media, including DMEM/F12 (Gibco, USA), B27 (Gibco, USA), basic fibroblast growth factor (bFGF, 20 ng/ml; AF-100-18B, PeproTech), and recombinant human epidermal growth factor (rhEGF, 20 ng/ml; AF-100-15, PeproTech). 5 Gy irradiation (IR) was applied to the GSCs 13 times in order to screen the GSCs with radiotherapy resistance. Subsequently, cells differentiated from GSCs (U87DIFF, Diff), which inherited the radiotherapy resistance, were induced by using DMEM with 10% FBS, followed by the culture of cells selected from clonal communities. The Diff-Luc cell line was generated in our laboratory through transfection with a reporter gene encoding firefly luciferase, as previously reported. The U87, Diff, and Diff-Luc cells were cultured using DMEM supplemented with 10% FBS (Gibco, CA, USA), and were grown in a humidified atmosphere of 5% CO_2_ at 37 °C.

### Transcriptome sequencing

Total RNA was extracted from cell samples using Trizol reagent, followed by purification and DNase I treatment to eliminate genomic DNA contamination. RNA quality was assessed using an Agilent Bioanalyzer to ensure high integrity. RNA-Seq libraries were prepared, including mRNA enrichment using oligo(dT) magnetic beads, synthesis of first and second-strand cDNA, end repair, adapter ligation, and PCR amplification. The libraries were quantified, and sequencing was performed using the Illumina platform to achieve comprehensive transcriptome analysis.

### Western blotting

Total protein was extracted from cells using RIPA buffer (Beyotime, China) supplemented with protease and phosphatase inhibitors (Beyotime, China), followed by centrifugation to collect the supernatant. Protein concentration was quantified using a BCA assay (Beyotime, China), and 50-80 µg of protein was resolved by SDS-PAGE. Proteins were transferred to a PVDF membrane and blocked with 5% BSA in TBS-T for 1 h at room temperature. The membrane was incubated overnight at 4 °C with primary antibodies, followed by incubation with HRP-conjugated secondary antibodies. Protein bands were visualized using an enhanced chemiluminescence (ECL) detection system.

### RT-qPCR

Total RNA was extracted from cells using Trizol reagent (Takara, Japan) and treated with DNase I to remove genomic DNA contamination. Reverse transcription was conducted using the Takara reverse transcription kit to synthesize cDNA. RT-qPCR was performed on the Roche LightCycler 480 System using ChamQ Universal SYBR qPCR Master Mix (Vazyme, China). The thermal cycling conditions included initial denaturation at 95 °C for 3 min, followed by 40 cycles of 95 °C for 10 s and 60 °C for 30 s. Relative gene expression levels were calculated using the 2^−ΔΔCt^ method with normalization to an internal control.

### CCK-8 assays

Cell viability was assessed using the Cell Counting Kit-8 (CCK-8, Abbkine Scientific, China). U87 and Diff cells were seeded into 96-well plates at a density of 1 × 10³ cells/well and allowed to adhere overnight. After treatment with various drugs, 10 μL of CCK-8 solution was added to each well and incubated at 37 °C for an additional 0.5 h. Absorbance was measured at 450 nm using a microplate reader (Spark, TECAN). Dose-response curves were drawn using GraphPad Prism software (version 10.0).

### Dead/live staining

Cell death and viability were assessed using the Dead/Live Cell Staining Kit (Beyotime, China). Treated cells were washed with PBS and incubated with a staining solution containing calcein-AM and propidium iodide (PI) at 37 °C for 20 min in the dark, according to the manufacturer’s instructions. Fluorescence images were captured using a fluorescence microscope (ECLIPSE Ti2-E, Nikon). Calcein-AM signals (green) indicate live cells, while PI signals (red) mark dead cells.

### EdU staining

Cell proliferation was evaluated using the EdU Cell Proliferation Assay Kit (Beyotime, China). After treatment, cells were incubated with EdU solution at 37 °C for 10 h, fixed with 4% paraformaldehyde, and permeabilized with 0.3% Triton X-100. The incorporated EdU was visualized by reaction with azide-conjugated fluorescent dye, as per the manufacturer’s protocol. Nuclei were counterstained with DAPI, and images were acquired using a fluorescence microscope (ECLIPSE Ti2-E, Nikon).

### Neurosphere formation assay

Diff cells (1000 cells/well) were plated in ultra-low attachment 96-well plates (Corning) containing serum-free stem cell medium. Cultures were maintained for 14 days with medium replacement every 72 h. Before each medium change, cells were treated with iRGD-OB-LP using standardized protocols from prior experiments. Neurospheres ≥75 µm in diameter were quantified using bright-field microscopy.

### Soft agar colony formation assay

Anchorage-independent growth was assessed using 0.7% low-melting agarose (BioFroxx, #2276GR005) layered over 1.2% agar base in six-well plates. Diff cells (5000 cells/well) suspended in medium containing 0.35% agarose were plated atop the base layer. Cultures were maintained for 30 days with medium replacement every 72 h, preceded by iRGD-OB-LP treatment per established protocols. Colonies with a >50-μm diameter were counted after crystal violet staining.

### Establishment of an orthotopic glioblastoma model

Diff-Luc cell line was generated for orthotopic tumor development. To create an orthotopic GBM model, a cell suspension containing 1 × 10^5^ cells/µl was injected in a volume of 3 µl into the right hindbrain of 4- to 6-week-old female BALB/c nude mice at coordinates relative to the bregma as (2 mm, −2 mm). All mouse studies were performed with the approval of the Institutional Animal Ethics and Welfare Committee of Nanfang Hospital, Southern Medical University (No. IACUC-LAC-20240522-005). D-Luciferin potassium (15 mg/ml, MCE, China) was intraperitoneally injected into the tumor-bearing mice for monitoring tumor size using intravital imaging (Spectral Instruments Imaging, USA).

### In vitro targeting and antitumor assay

All liposomes were dissolved in OPTI-MEM (Gibco, USA), with the total lipid concentration serving as a proxy for liposome concentration. Cells were exposed to varying liposome concentrations for a defined period. Post-treatment, the solution was aspirated, and cells were gently rinsed with PBS. Subsequently, cells were cultured in DMEM supplemented with 10% FBS for 24 h before commencing subsequent experimental procedures. Flow cytometry (CytoFLEX, Beckman Coulter, USA) was employed to assess the cellular uptake efficiency of different liposome formulations.

### In vivo targeting and antitumor assay

For in vivo tumor targeting, orthotopic GBM models were established as previously described [31]. Following tumor cell implantation and confirmation of tumor formation via bioluminescence imaging (BLI), mice were randomized into groups. Gradient doses (30, 60, and 90 μL/10 g body weight) of DID-labeled liposomes were intravenously administered via the tail vein. Fluorescence imaging was performed at 12, 24, 36, and 48 h post-injection using an intravital imaging system (Spectral Instruments Imaging, USA) to assess tumor-specific accumulation. Additionally, a subset of mice was sacrificed at 24 h post-injection for ex vivo imaging to evaluate the biodistribution of liposomes in major organs.

For the antitumor efficacy assay, orthotopic GBM tumors were confirmed by BLI. Mice were then randomized into groups and treated with either normal saline, a combination of free Osimertinib and Bortezomib, iRGD-LP, Non-OB-LP, or iRGD-OB-LP. Treatments were administered via tail vein injection every 48 h. Radiotherapy (2 Gy per session, PXi X-RAD225) was initiated 24 h after the first dose of drug administration and continued every 24 h for a total of five sessions, completing one treatment cycle. Two treatment cycles were conducted in total. Tumor growth was monitored through BLI before treatment initiation, midway, and at the end of the treatment cycles. Tumor size was quantitatively analyzed based on fluorescence intensity. Survival analysis was conducted using the Kaplan–Meier method.

### In vivo safety assessment

Healthy 4–6-week-old female nude mice were intravenously administered saline, Non-LP, or iRGD-LP every 48 h for a total of five doses. Following treatment, whole blood was collected into anticoagulant tubes and analyzed using a hematology analyzer and a biochemical analyzer. The following parameters were assessed: white blood cell count (WBC), red blood cell count (RBC), platelet count (PTL), hemoglobin level (HGB), alanine aminotransferase (ALT), aspartate aminotransferase (AST), blood urea nitrogen (BUN), and creatinine (Cre).

### Hemolysis experiment

SD rats were anesthetized, and blood was harvested from the abdominal aorta into anticoagulant tubes. Following centrifugation to remove the plasma, the erythrocyte pellet was collected. A total of 20 µL of the blood cells was then mixed with 1 mL of Non-LP and iRGD-LP solutions at varying concentrations and incubated at 37 °C for 4 h. After centrifugation at 1500 rpm for 15 min, the supernatant was discarded, and the hemolytic effect of the liposomes was assessed by capturing photographic images of the resulting hemolysis.

### Immunofluorescence (IF) staining

For immunofluorescence staining, cells were seeded on glass coverslips, fixed with 4% paraformaldehyde for 15 min, and permeabilized with 0.3% Triton X-100 for 10 min. After blocking with 5% bovine serum albumin (BSA) for 1 h, cells were incubated with primary antibodies overnight at 4 °C. Coverslips were washed with PBS and incubated with fluorophore-conjugated secondary antibodies for 1 h at room temperature in the dark. Nuclei were counterstained with DAPI, and samples were imaged using a fluorescence microscope (ECLIPSE Ti2-E, Nikon).

### Immunohistochemistry (IHC) staining

Tissue sections were deparaffinized in xylene and rehydrated through a graded ethanol series. Antigen retrieval was performed using EDTA buffer (pH 9.0). Sections were blocked with 5% BSA for 30 min and incubated with primary antibodies overnight at 4 °C. Following washes, sections were incubated with HRP-conjugated secondary antibodies (ZSGB-Bio, China) for 1 h at room temperature. The signal was developed using a DAB substrate kit (ZSGB-Bio, China), and nuclei were counterstained with hematoxylin.

### Hematoxylin–eosin (H&E) staining

Tissue samples were first fixed in 4% paraformaldehyde for 24 h and then embedded in paraffin. Sections of 4–5 µm thickness were cut and mounted onto glass slides, followed by deparaffinization in xylene and rehydration through graded alcohols (100, 95, and 70%). The slides were then stained with hematoxylin for 8 min, rinsed with water, and differentiated in 1% acid alcohol. After a thorough rinse, the slides were stained with eosin for 5 min, dehydrated in alcohol, cleared in xylene, and mounted for observation under a microscope.

## Results

### Establishment and functional profiling of radioresistant GBM cell models

**Fig. 1:**
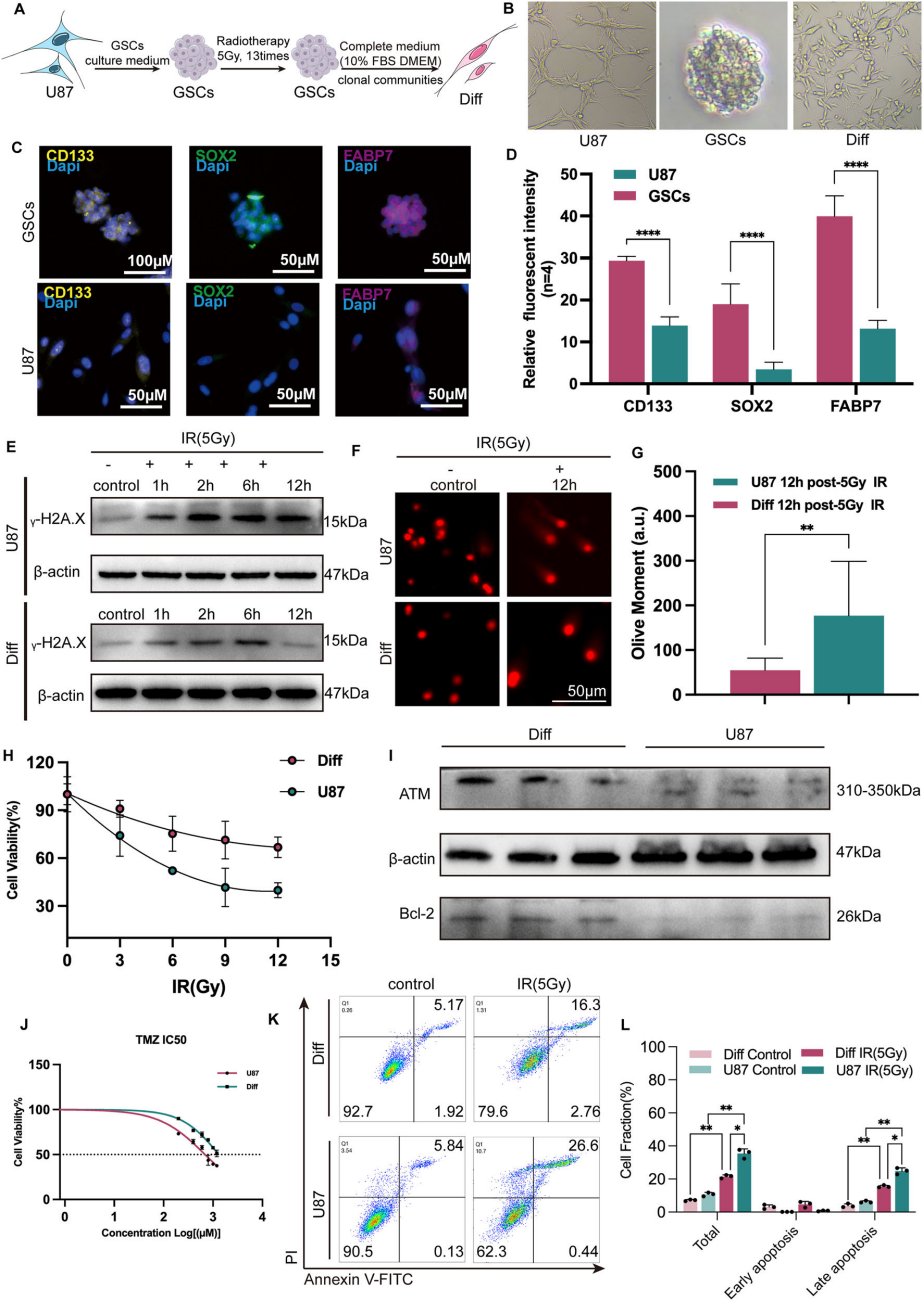
Culture and characterization of radioresistant GBM cell lines. **A** Schematic diagram illustrating the culture process for generating radioresistant GBM cell lines. **B** Light microscopy images showing the morphology of three GBM cell lines. **C**, **D** Fluorescence images depicting the expression of stemness markers (CD133, SOX2, FABP7) in GSCs and U87 cells. ****p < 0.0001. **E** Western blot analysis of ***γ***H2AX expression in U87 and Diff cells at multiple time points (1, 2, 6, and 12 h) after 5 Gy irradiation. **F**, **G** Comet gel electrophoresis assay results in U87 and Diff cells at 12 h after 5 Gy irradiation. Representative images and quantitative analysis of Olive Moment (I). **p < 0.01. **H** Comparison of cell viability in U87 and Diff cells following different irradiation doses. **I** Western blot analysis showing elevated expression of ATM and Bcl-2 in Diff cells. **J** Dose-response curve for TMZ treatment in U87 and Diff cells. **K**, **L** Apoptosis rates of U87 and Diff cells 72 h post-5Gy irradiation treatment. **p < 0.01, *p < 0.05.

To investigate the mechanisms underlying glioblastoma radioresistance, we established a progressive irradiation model using U87-derived glioblastoma stem cells (GSCs). Through 13 cycles of 5 Gy irradiation (cumulative dose: 65 Gy). We successfully induced radioresistant GSCs that maintained stemness properties, as confirmed by sustained CD133, SOX2, and FABP7 expression through immunofluorescence analysis (Fig. 1A–D). These radioresistant GSCs exhibited predominant mesenchymal (MES) characteristics over proneural (PN) features, supported by ssGSEA scoring of signature gene expression patterns [32] (Fig. S1C–E). Subsequent differentiation of GSCs generated radiation-adapted differentiated cells (Diff cells), completing the cell model triad: parental U87, GSCs, and Diff cells (Fig. 1A, B).

Principal component analysis (PCA) revealed distinct transcriptomic landscapes among the three cell types, with clear spatial separation along principal component axes 1 and 2, indicative of their divergent molecular identities (Fig. S1A). Remarkably, Diff cells exhibited pronounced neuronal morphological features (Fig. S1B), demonstrating the retained differentiation plasticity of radioresistant GSCs.

Functional characterization demonstrated that Diff cells acquired enhanced radioresistance compared to parental U87 cells, manifested through: increased post-irradiation viability (Fig. 1H), suppressed radiation-induced apoptosis (Fig. 1K, L) and accelerated DNA repair capacity (Fig. 1E–G) evidenced by 2.2-fold lower residual DNA damage in Diff cells (median olive moment = 56.7 vs U87 = 125.4; *p* < 0.001) at 12 h post-5Gy irradiation. Mechanistically, Diff cells exhibited elevated ATM expression—a key regulator of DNA damage response—accompanied by upregulation of the anti-apoptotic protein Bcl-2 (Fig. 1I), providing a molecular basis for their enhanced DNA repair efficiency and apoptosis resistance. Notably, Diff cells also developed cross-resistance to temozolomide (Fig. 1J), suggesting broad-spectrum therapy resistance and also mirroring clinical GBM recurrence profiles.

### Bortezomib and osimertinib as potential radiosensitizers for glioblastoma

**Fig. 2:**
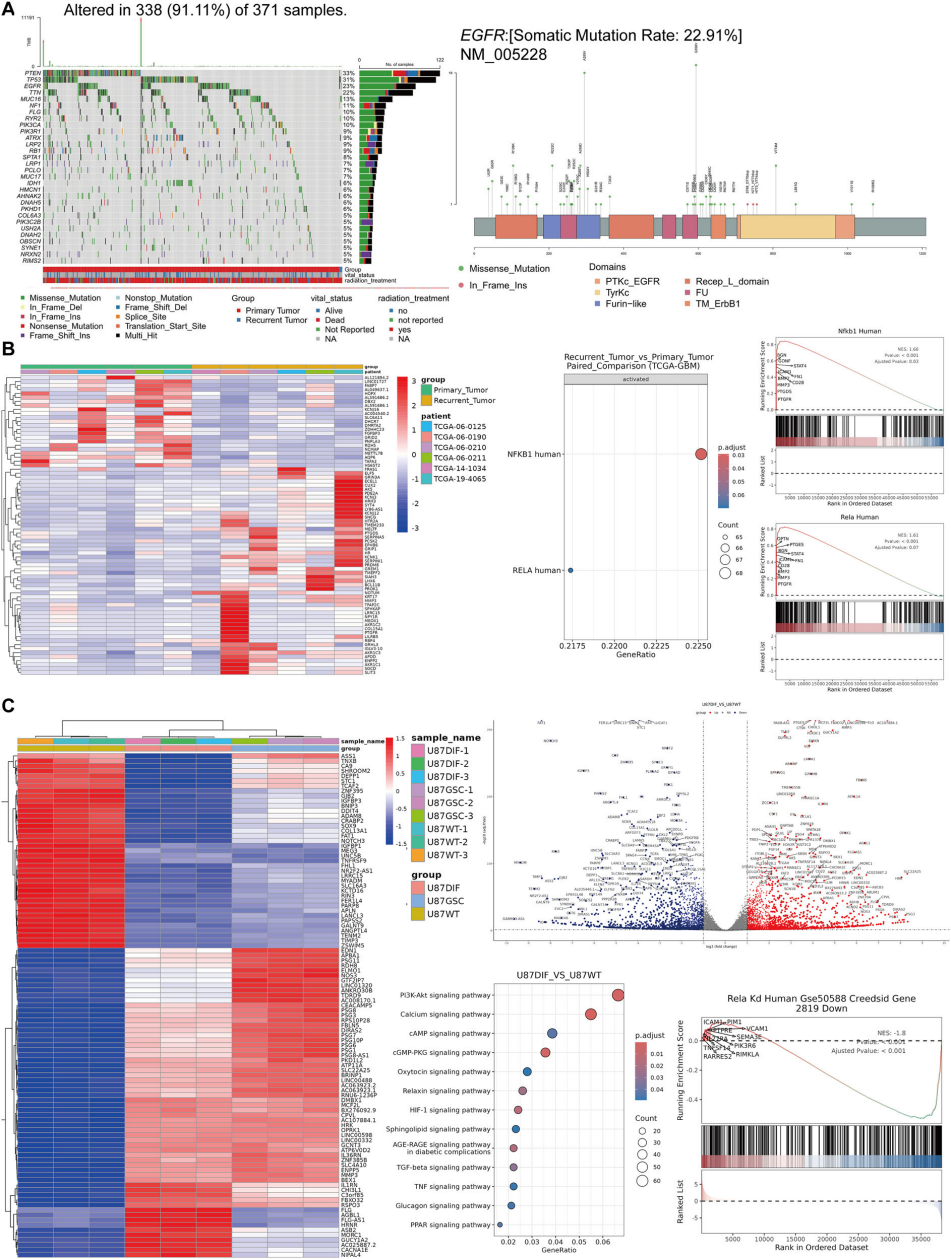
Analysis of TCGA GBM samples and RNA-seq analysis of radioresistant GBM cell lines. **A** Gene mutation waterfall plot of TCGA GBM samples, displaying mutation frequencies, and a lollipop plot indicating specific EGFR mutation sites. **B** Heatmap depicting differential gene expression profiles between primary and recurrent GBM tissues. GSEA analysis highlights significant activation of the NF-κB signaling pathway, particularly its key transcription factor subunits RELA/P65 and NFKB1/P50, in recurrent GBM. **C** Heatmap and volcano plot showing differential gene expression among U87DIFF (Diff) cells, U87GSCs (GSCs), and U87WT (U87) cells. Dot plots from GSEA analysis highlight transcription factor binding activity and pathway activation, including the EGFR/PI3K/AKT and NF-κB pathways, particularly in radioresistant Diff cells.

Receptor tyrosine kinases (RTKs) are frequently dysregulated in cancer, with aberrant signaling driving tumorigenesis and progression. Among these, the ErbB family plays a critical role in transducing extracellular signals and activating downstream RTK-RAS signaling cascades via effector pathways. EGFR, a key ErbB member, is central to tumor development and radioresistance. Analysis of TCGA data reveals genetic alterations in 91.11% (338/371) of GBM cases, with EGFR mutations ranking third in frequency (22.91%; Fig. 2A). EGFR-mediated radioresistance has been widely studied in various tumors, including GBM. Mechanistically, EGFR can promote tumor radioresistance through multiple signaling pathways, including the RAS/RAF/MAPK and PI3K/AKT pathways [33–37]. To counteract this, we selected Osimertinib, a potent EGFR inhibitor, to disrupt EGFR activation, offering a promising strategy to overcome radioresistance and improve therapeutic outcomes.

RNA transcriptome sequencing analysis of TCGA data identified distinct gene expression profiles between primary and recurrent GBM tissues (Fig. 2B). Notably, Gene Set Enrichment Analysis (GSEA) revealed significant upregulation of the NF-κB signaling pathway in recurrent tumors, with pronounced activation of its transcription factors RELA/P65 and NFKB1/P50 (Fig. 2B). This underscores NF-κB’s role in promoting recurrence and therapeutic resistance. To target this pathway, we employed Bortezomib, a proteasome inhibitor that effectively suppresses NF-κB activation, as a therapeutic candidate.

To determine whether the activation of the EGFR and NF-κB signaling pathways, as observed in the TCGA data, also occurs in our constructed radiotherapy-resistant cell lines, we performed whole transcriptome sequencing on the U87WT (U87), U87GSC (GSCs), and U87DIFF (Diff) cell lines. The results (Fig. 2C) revealed distinct gene expression profiles and, crucially, significant activation of the EGFR/PI3K/AKT and NF-κB pathways specifically in U87DIFF cells. This mirrors the TCGA findings, confirming that our U87DIFF cells effectively model clinical radiotherapy resistance and recurrence. This process parallels the clinical scenario where residual GSCs surviving surgery and radiotherapy evolve into resistant stem cells, ultimately generating heterogeneous, treatment-resistant tumor populations.

These findings validate EGFR and NF-κB as critical mediators of GBM radioresistance. Prior to liposomal drug formulation, we assessed the therapeutic synergy of Osimertinib and Bortezomib. Combination Index (CI) analysis demonstrated additive to synergistic effects, while apoptosis assays revealed significantly enhanced cell death, particularly in radioresistant Diff cells (Fig. S2A, B). This confirms the dual-targeting strategy’s efficacy in overcoming radioresistance and underscores the necessity of EGFR and NF-κB signaling for survival in therapeutically resistant GBM.

### Preparation and characterization of liposomes

**Fig. 3:**
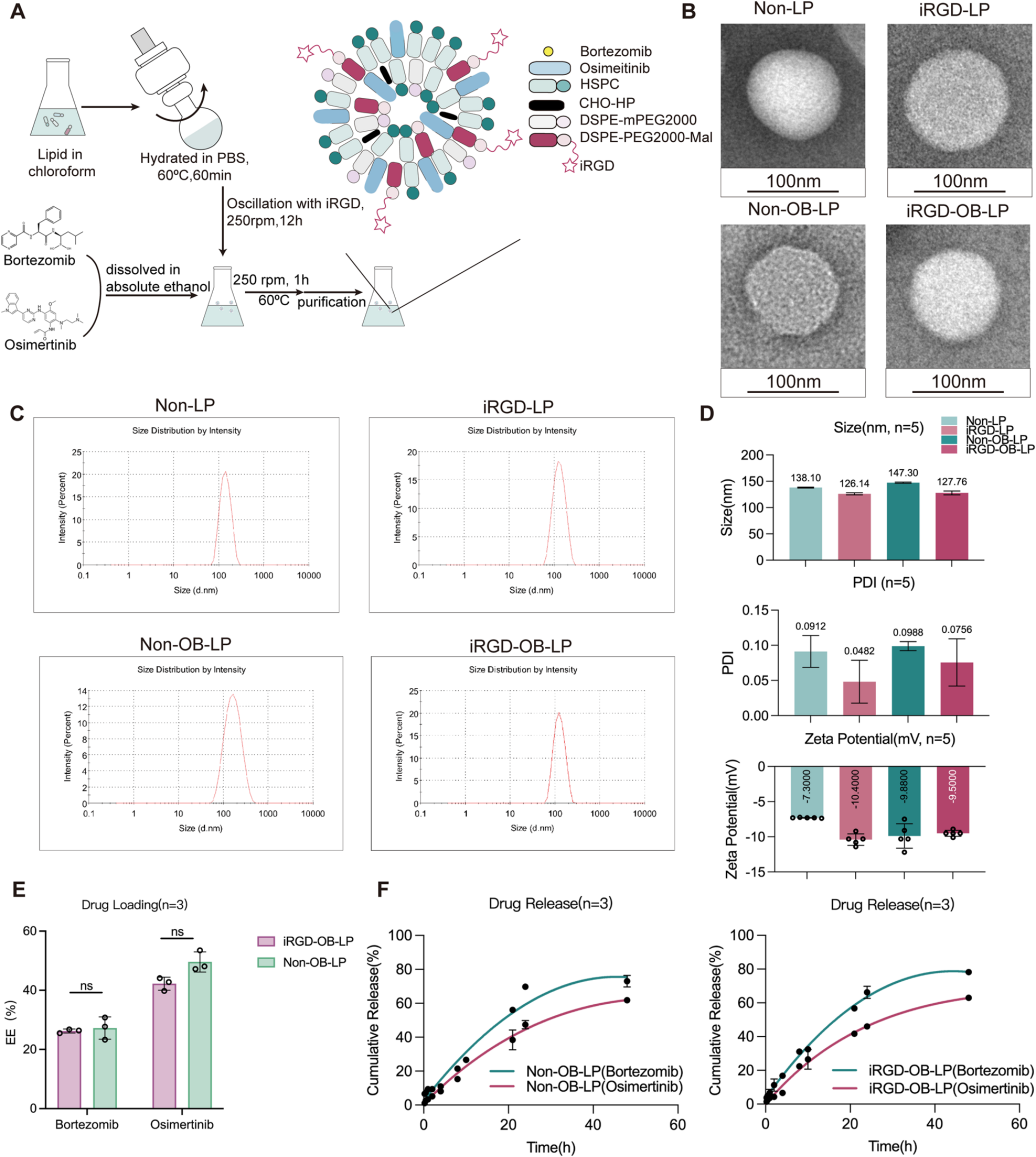
Liposome synthesis, validation, characterization, and evaluation. **A** Schematic diagram of the iRGD-OB-LP showing iRGD, bortezomib, osimertinib, and liposomes with lipid materials (HSPC, CHO-HP, mPEG2000, and DSPE-PEG2000-MAL). **B** TEM images of Non-LP, iRGD-LP, Non-OB-LP, and iRGD-OB-LP showing cloudy liposomal coatings around dark cores. Scale bar: 100 nm. **C** The hydrodynamic diameter of non-LP, iRGD-LP, non-OB-LP, and iRGD-OB-LP. **D** The physicochemical properties of liposomes (*n* = 5). **E** EE% of bortezomib and osimertinib of non-OB-LP and iRGD-OB-LP (*n* = 3). **F** Drugs in vitro release pattern of non-OB-LP and iRGD-OB-LP. Under simulated in vivo conditions, a slow release was observed over 48 h, with no apparent burst release effect.

Liposomes without iRGD (non-targeted liposomes, non-LP) and iRGD-modified liposomes (iRGD-LP) were fabricated via the film dispersion method. Both formulations encapsulated Bortezomib and Osimertinib, yielding non-OB-LP and iRGD-OB-LP (Fig. 3A). Transmission electron microscopy (TEM) images confirmed uniform spherical morphology for all liposomes, with a distinct hydrated iRGD-PEG outer layer (Fig. 3B and S2C). Physicochemical characterization by dynamic light scattering (DLS) demonstrated narrow size distributions, with hydrodynamic diameters of approximately 125–150 nm and polydispersity index (PDI) values below 0.1 (Fig. 3C, D). Zeta potential measurements revealed negative surface charges between −7.3 and −10.3 mV (Fig. 3D).

Entrapment efficiency (EE%) was evaluated by disrupting purified liposomes with acetonitrile and quantifying the supernatant absorbance via UV-Vis spectrophotometry (Fig. S2E). Osimertinib exhibited EE% values of 42–50%, whereas bortezomib displayed lower entrapment (25–28%). iRGD modification showed no significant effect on EE% (Fig. 3E). UV-Vis spectrophotometric analysis of the dialysate also revealed a conjugation efficiency exceeding 90% for iRGD linked to liposomes (Fig. S2F).

For drug release profiling, liposomes in dialysis bags were incubated in PBS containing 10% FBS at 37 °C with agitation (80 rpm) for 48 h. Both formulations exhibited sustained release kinetics without an initial burst, and no statistically significant differences were observed between Non-OB-LP and iRGD-OB-LP (Fig. 3F). Furthermore, CCK-8 assays confirmed negligible cytotoxicity for iRGD-LP, indicating favorable biocompatibility (Fig. S2D).

### Cellular uptake and lysosomal escape of liposomes

Both non-LP and iRGD-LP were labeled with FITC, and flow cytometry (FCM) was used to assess their uptake rates of these liposomes in U87 and Diff cells, both of which highly express integrin αvβ3 (CD51/CD61) and NRP1 (CD304), consistent with the expression levels observed in primary and recurrent GBM from TCGA data (Fig. S3A, B).

**Fig. 4:**
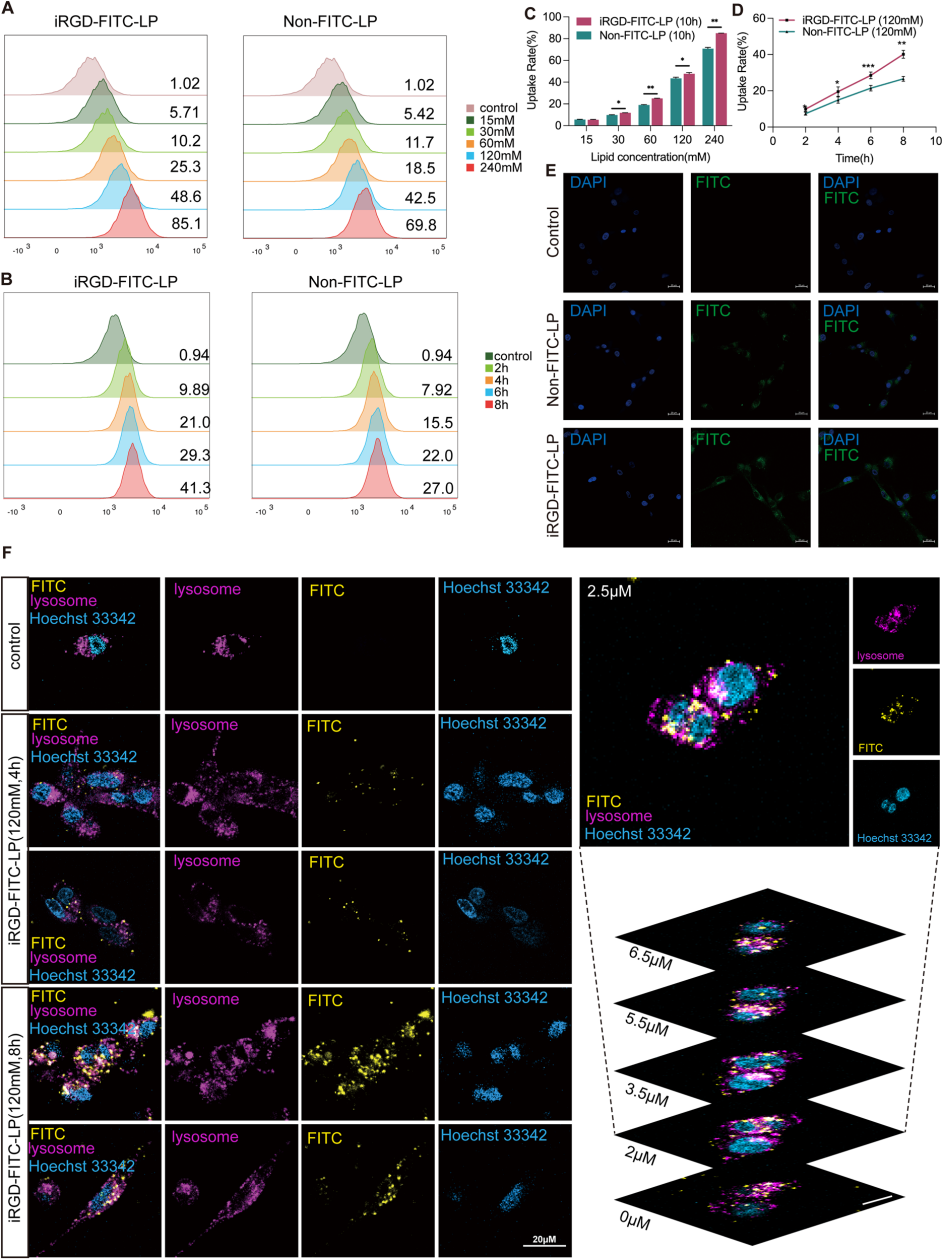
In vitro liposome uptake by GBM cells. **A** Flow cytometry showing the uptake rates of Non-FITC-LP and iRGD-FITC-LP in U87 cells at varying concentrations after incubating for 10 hours. **B** Flow cytometry histograms (FITC channel) demonstrating the uptake rates of Non-FITC-LP and iRGD-FITC-LP in U87 cells at the same concentration(120 mM) over different time intervals. (**C, D**) Quantitative analysis of cellular uptake of Non-FITC-LP and iRGD-FITC-LP. Statistical significance: **p < 0.01, ***p < 0.001. **E** Confocal microscopy images illustrating cellular localization of iRGD-FITC-LP (green) and nuclei stained with DAPI (blue). Scale bar: 20 µm. **F** Confocal microscopy images showing colocalization of lysosomes (red, Lysotracker Red DND-99) and iRGD-FITC-LP (yellow, FITC) in Diff cells. Scale bar: 20 µm.

Liposomes were dispersed in Opti-MEM and incubated with cells for 10 h. The results showed that cellular uptake rates increased with higher liposome concentrations, with consistently higher uptake observed for iRGD-FITC-LP compared to non-FITC-LP (Fig. 4A, C and Fig. S3C). These findings were further supported by confocal microscopy images, which demonstrated greater uptake of iRGD-FITC-LP by the U87 cells under identical conditions (Fig. 4E). Additionally, the uptake rates were time-dependent, increasing with longer incubation times. At the same lipid concentration (120 mM), the iRGD-FITC-LP group maintained significantly higher uptake rates compared to the non-FITC-LP group at all tested time points (Fig. 4B, D).

To assess lysosomal escape, Lysotracker Red DND-99 was used for lysosome staining, and Hoechst 33342 for nuclear staining. Colocalization analysis revealed that while some iRGD-FITC-LP co-localized with lysosomes, the majority remained dispersed in the cytoplasm (Fig. 4F and S3D). This finding suggests that iRGD-modified liposomes possess effective lysosomal escape, thereby protecting the encapsulated drugs from lysosomal enzymatic degradation.

### Enhancing radiosensitivity with liposomes: effects on cell viability, apoptosis and proliferation in vitro

To evaluate the antitumor and radiosensitizing effects of liposomes, we dispersed the formulations in Opti-MEM and treated cells for 10 h. Post-treatment, the medium was replaced with DMEM supplemented with 10% FBS, and subsequent experiments were performed after 24 h of standard culture.

**Fig. 5:**
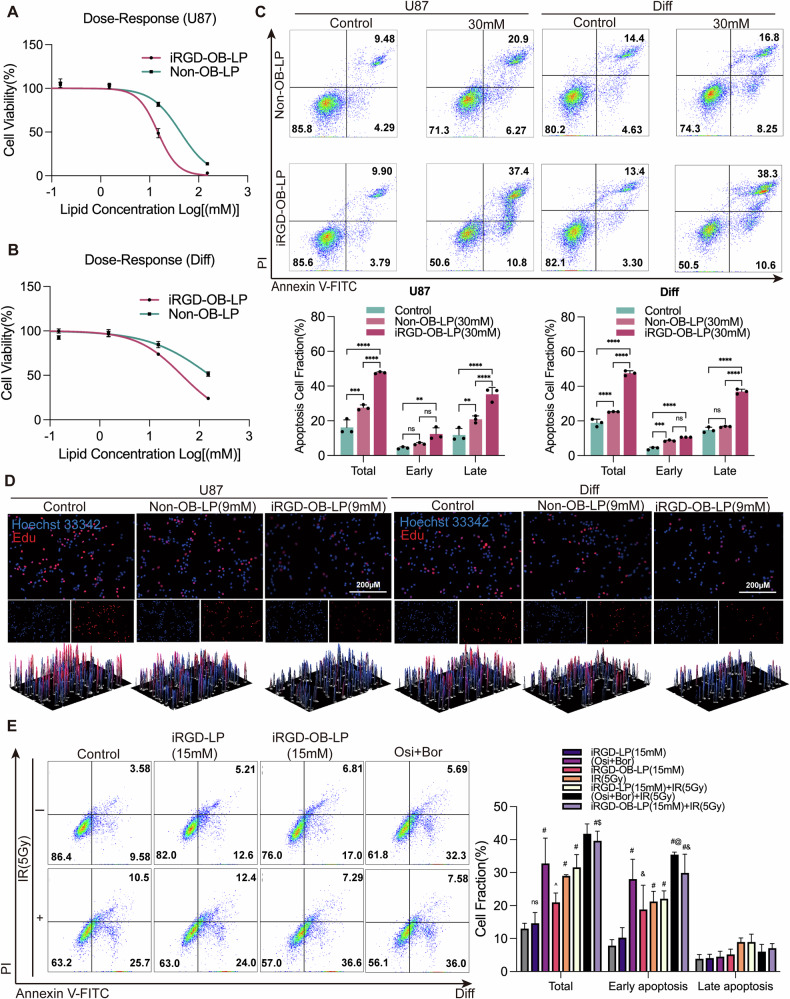
In vitro antitumor and radiosensitization effects. **A**, **B** Dose-response curves for iRGD-OB-LP and non-OB-LP in U87 and Diff cells. **C** Apoptosis analysis of U87 and Diff cells after 72 h post-treatment with iRGD-OB-LP and non-OB-LP, along with quantitative analysis of apoptosis rates. Statistical significance: **p < 0.01, ****p < 0.0001. **D** EdU staining analysis showing the proliferative effects of iRGD-OB-LP and non-OB-LP on U87 and Diff cells. **E** Apoptosis analysis of Diff cells treated with iRGD-LP, iRGD-OB-LP, free osimertinib and bortezomib (in combination), with or without 5 Gy irradiation. Statistical comparisons: # compared with control (-IR), *p* < 0.0001; $ compared with control (+IR), *p* < 0.01; @ compared with control (+IR), *p* < 0.001; & compared with control (-IR), *p* < 0.01; ^ compared with control (-IR), *p* < 0.05.

Dose-response analyses via CCK-8 assays revealed that both U87 and Diff cells demonstrated greater sensitivity to iRGD-OB-LP compared to non-OB-LP, as indicated by significantly lower IC50 values (Fig. 5A, B). Furthermore, EdU staining, live/dead assays, and CCK-8 results demonstrated that even at low concentrations (9 mM), iRGD-OB-LP significantly suppressed proliferation and induced cell death, whereas Non-OB-LP showed no significant effects (Fig. 5D and S4A, D).

Apoptosis assays corroborated these findings, with iRGD-OB-LP inducing markedly higher apoptosis rates in both cell lines compared to non-OB-LP (*p* < 0.0001), particularly in late-stage apoptosis (Fig. 5C). Notably, increasing iRGD-OB-LP concentrations from 30 to 60 mM did not produce a significant increase in apoptosis (Fig. S4B).

Radiotherapy sensitization assays further confirmed that iRGD-OB-LP enhanced radiation-induced apoptosis in Diff cells (Fig. 5E). Treatments with iRGD-LP, iRGD-OB-LP, and the bortezomib-osimertinib positive control induced distinct apoptosis levels across experimental groups. Following irradiation, however, only iRGD-OB-LP and the bortezomib-osimertinib combination significantly amplified radiotherapy efficacy (*p* < 0.0001).

### iRGD-OB-LP effectively inhibits EGFR and NF-κB signaling pathways to enhance radiosensitivity in GBM

**Fig. 6:**
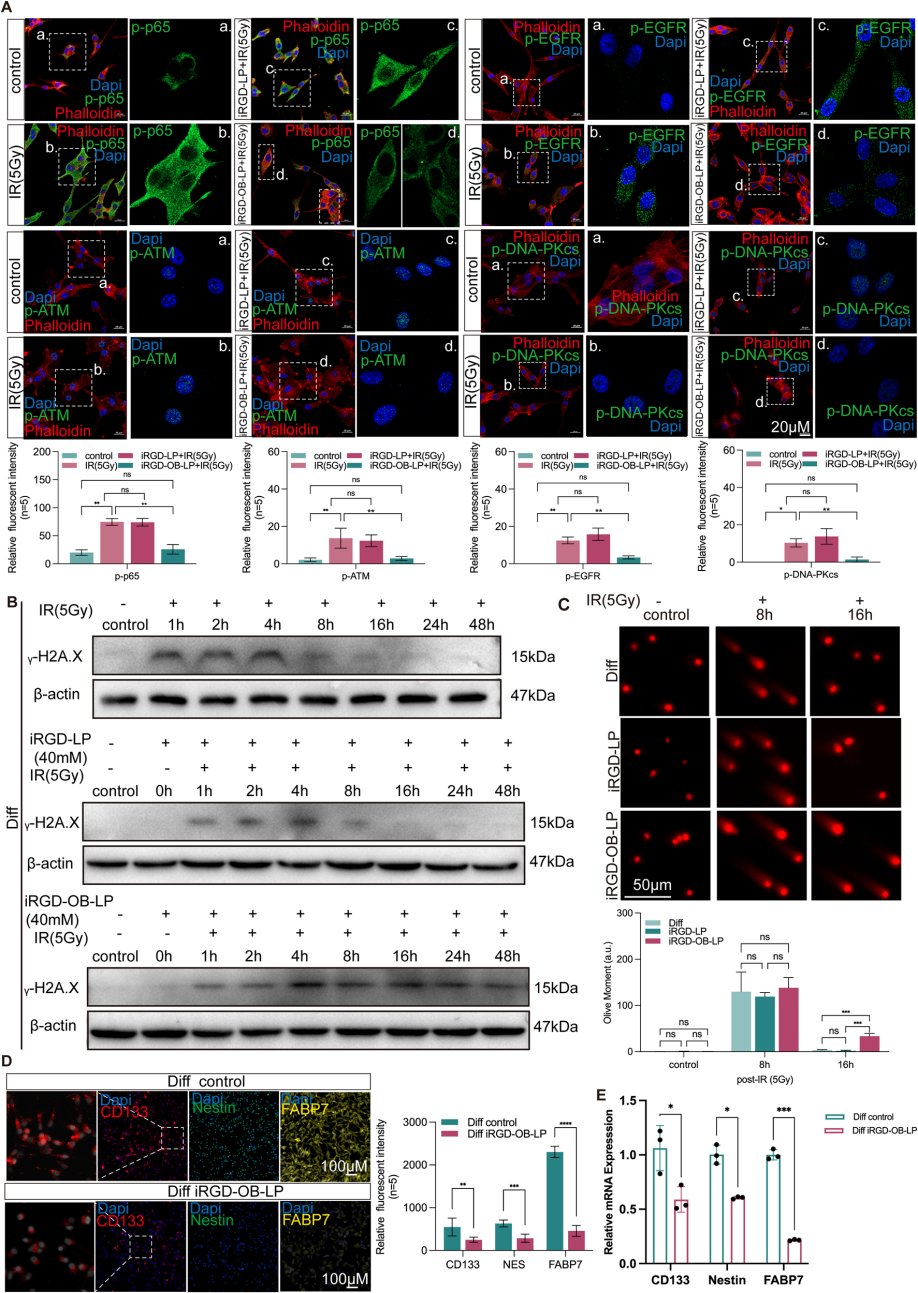
iRGD-OB-LP effectively inhibits EGFR and NF-κB signaling pathways and alters stemness in Diff cells. **A** Fluorescence images showing that iRGD-OB-LP treatment significantly inhibits the phosphorylation and activation of p65, ATM, EGFR, and DNA-PKcs at 1–2 h post-5 Gy irradiation in Diff cells. Statistical significance: *p < 0.05, **p < 0.01. **B** Persistent evaluation of γ-H2AX expression in Diff cells at various time points (0, 1, 2, 4, 8, 12, 24, and 48 h) after 5 Gy irradiation, with treatments including iRGD-LP, iRGD-OB-LP, or no drug (control). **C** Comet gel electrophoresis assay results in Diff cells at 8 or 16 h after 5 Gy irradiation, with treatments including iRGD-LP, iRGD-OB-LP, or no drug (control) and quantitative analysis of olive moment. Statistical significance: ***p < 0.001. **D**, **E** Fluorescence images and RT-qPCR analysis of the expression of stemness markers (CD133, Nestin, and FABP7) in Diff cells treated with iRGD-OB-LP or left untreated. Statistical significance: *p < 0.05, **p < 0.01,***p < 0.001, ****p < 0.0001.

To investigate the mechanism underlying iRGD-OB-LP-mediated enhancement of radiosensitivity, Diff cells were incubated with iRGD-OB-LP for 10 h, followed by replacement with DMEM containing 10% FBS for 24 h, and subsequently irradiated with 5 Gy. Light microscopy (Fig. S4C) revealed that iRGD-OB-LP induced pronounced cell rounding and vacuolization, effects further amplified by 5 Gy irradiation. Confocal microscopy demonstrated marked suppression of EGFR and NF-κB signaling pathway activation within 1–2 h post-irradiation in iRGD-OB-LP-treated Diff cells. Additionally, downstream DNA damage repair mediators, including phosphorylated ATM (p-ATM) and DNA-PKcs (p-DNA-PKcs), were significantly inhibited (Fig. 6A). Concurrently, iRGD-OB-LP suppressed radiation-induced phosphorylation of AKT (pAKT) and reduced expression of the anti-apoptotic protein Bcl-2 (Fig. S5A, B). These findings indicate that 5 Gy irradiation activates EGFR and NF-κB signaling, which promotes DNA double-strand break repair via nonhomologous end joining (NHEJ; via DNA-PKcs phosphorylation) and homologous recombination (HR; via ATM phosphorylation). However, iRGD-OB-LP treatment suppresses both pathways, abolishing DNA-PKcs and ATM phosphorylation, thereby inhibiting NHEJ and HR repair. This impairment of DNA repair mechanisms exacerbates radiation-induced DNA damage in GBM cells, enhancing radiosensitivity.

Western blot analysis of γ-H2AX demonstrated that iRGD-OB-LP combined with 5 Gy irradiation resulted in persistent DNA damage, as evidenced by sustained γ-H2AX expression for up to 48 h. In contrast, untreated cells or those pre-treated with iRGD-LP alone efficiently repaired DNA damage within ~8 h post-irradiation (Fig. 6B, C), further supporting iRGD-OB-LP’s role in suppressing DNA repair. Furthermore, iRGD-OB-LP treatment significantly reduced mRNA expression of the radioprotective cytokine IL-6 and Bcl-2 (Fig. S5B), reinforcing its broader suppression of pro-survival signaling post-radiation.

Interestingly, the expression of classical stemness markers such as CD133 and Nestin was significantly reduced in Diff cells after treatment with iRGD-OB-LP, as confirmed by both IF staining and RT-qPCR analysis (Fig. 6D, E). Critically, this molecular-level reduction in stemness was corroborated by functional assays: iRGD-OB-LP treatment markedly decreased tumorsphere formation capacity and suppressed colony growth in soft agar (Fig. S6A, B), directly demonstrating impaired self-renewal and tumorigenic potential. Collectively, these findings indicate that iRGD-OB-LP not only enhances radiosensitivity but also potently impairs the tumor’s self-renewal, resistance, and malignant capabilities.

In addition, FABP7, a brain-specific fatty acid-binding protein, was markedly downregulated following iRGD-OB-LP treatment. This reduction in FABP7 was accompanied by significant alterations in lipid metabolism. Specifically, RT-qPCR analysis revealed concomitant downregulation of key lipid metabolism regulators, including the master transcriptional regulator PPARγ as well as FABP4, PLIN2, and CD36 (Fig. S6D, E), collectively indicating a broad suppression of lipid metabolic pathways. Bodipy staining revealed a notable reduction in intracellular lipid droplet content in Diff cells treated with iRGD-OB-LP. Since lipid droplets are known to protect against radiotherapy by reducing the oxidation of polyunsaturated fatty acids (PUFAs), which is caused by ROS [38], we assessed ferroptosis modulation. Ferroptosis markers PTGS2 and TFRC were upregulated in iRGD-OB-LP-treated Diff cells, indicating heightened ferroptosis susceptibility. Furthermore, iRGD-OB-LP increased ROS generation in post-radiotherapy Diff cells, a central mediator of ferroptosis (Fig. S6D, E), accompanied by a significant increase in malondialdehyde (MDA), a key end-product of lipid peroxidation and a definitive marker of ferroptosis execution (Fig. S6H).

Collectively, these results demonstrate that iRGD-OB-LP enhances radiosensitivity by impairing DNA repair, reducing stemness, and disrupting lipid metabolism in Diff cells. This dual mechanism of sustaining DNA damage while inhibiting protective pathways, including ferroptosis resistance, highlights iRGD-OB-LP’s therapeutic potential for glioblastoma treatment.

### Distribution and tumor targeting of iRGD-modified liposomes in vivo

To investigate the biodistribution and intracranial tumor penetration of candidate liposomes in vivo, an orthotopic glioblastoma xenograft model expressing a luciferase reporter gene (Diff-Luc) was established. In this model, 3 µl of a cell suspension (1 × 10^5^ cells/µl) was stereotactically injected into the right hindbrain of 4- to 6-week-old female BALB/c nude mice. Tumor formation was confirmed 21 days later using intravital imaging, enabling the evaluation of liposome delivery to the tumor site. Before in vivo administration, the safety profile of the liposomes was evaluated, demonstrating no significant toxicity (Fig. S7A–C).

**Fig. 7:**
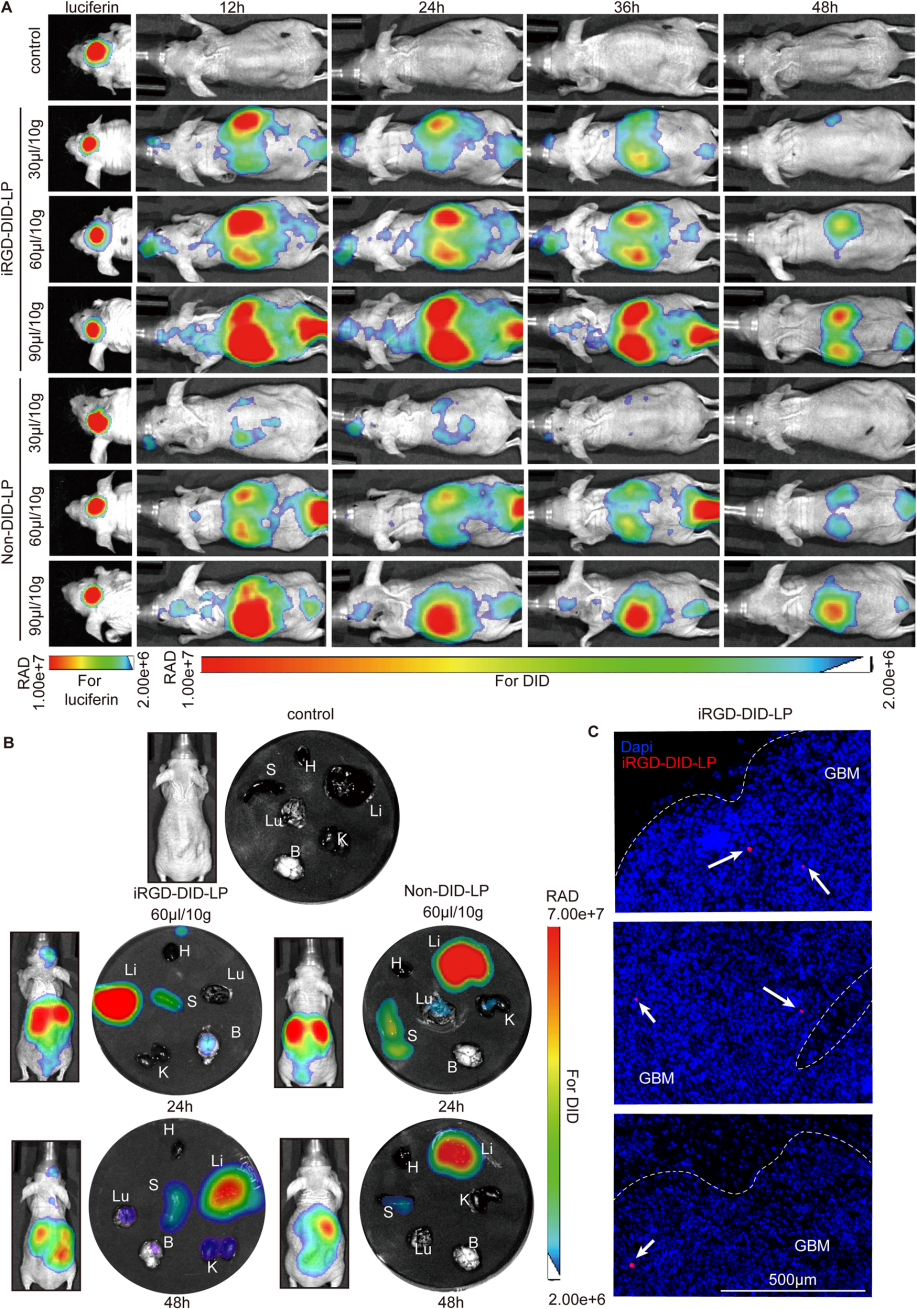
iRGD-modified liposome targets GBM in vivo. **A** Intravital imaging of tumor-bearing mice showing the biodistribution of iRGD-DID-LP and non-DID-LP at varying concentrations (30 µl/10 g, 60 µl/10 g, and 90 µl/10 g) and time points (12, 24, 36, and 48 h). **B** In vivo and ex vivo fluorescence imaging depicting the distribution of iRGD-DID-LP and non-DID-LP in various organs, including the brain (B), heart (H), liver (Li), spleen (S), lungs (Lu), and kidneys (K), at 24 and 48 h post-injection. **C** Fluorescence images of iRGD-DID-LP distribution in the brain. The dashed line indicates the region of GBM. Nuclei are stained in blue, and iRGD-DID-LP is shown in red. Scale bar: 500 µm.

To study liposome biodistribution, iRGD-LP and Non-LP were labeled with DID dye to allow fluorescent tracking. Mice were treated with different concentrations of iRGD-DID-LP and Non-DID-LP via tail vein injection. Intravital imaging revealed that iRGD-DID-LP effectively accumulated in the head region, particularly at doses of 60 µl/10 g and 90 µl/10 g, while mice treated with Non-DID-LP exhibited no significant accumulation. Notably, the fluorescent signal from iRGD-DID-LP in the head region disappeared 48 h post-treatment, suggesting that the liposomes were metabolized (Fig. 7A). Based on these findings, a dosing schedule of 60 µl/10 g every 48 h was selected for further experiments.

To further examine the in vivo distribution of liposomes, mice were euthanized 24 or 48 h after treatment for ex vivo organ fluorescence imaging. The images revealed that the majority of the liposomes accumulated in the liver and spleen, with moderate distribution to the lungs and kidneys. However, importantly, 24 h post-treatment, iRGD-DID-LP was distinctly detected in the tumor area, a distribution pattern that was not observed with non-DID-LP (Fig. 7B).

To confirm tumor targeting at the cellular level, tumor-bearing mice were euthanized 24 h after iRGD-DID-LP administration, and their brain tissues were harvested for frozen sectioning. Fluorescence microscopy of the tumor sections revealed the presence of iRGD-DID-LP within the tumor tissue, further corroborating the ability of the iRGD modification to penetrate the blood–brain barrier and selectively accumulate within glioblastoma tissue (Fig. 7C).

These findings demonstrate that iRGD-modified liposomes achieve targeted GBM accumulation in vivo. Rapid systemic clearance and metabolic elimination within 48 h support a repeated dosing strategy for sustained therapeutic efficacy. The tumor-specific distribution of iRGD-DID-LP, absent in non-DID-LP, highlights the critical role of iRGD modification in enhancing glioblastoma-targeted delivery.

### In vivo evaluation of iRGD-modified liposomes as radiosensitizers for radioresistant GBM

**Fig. 8:**
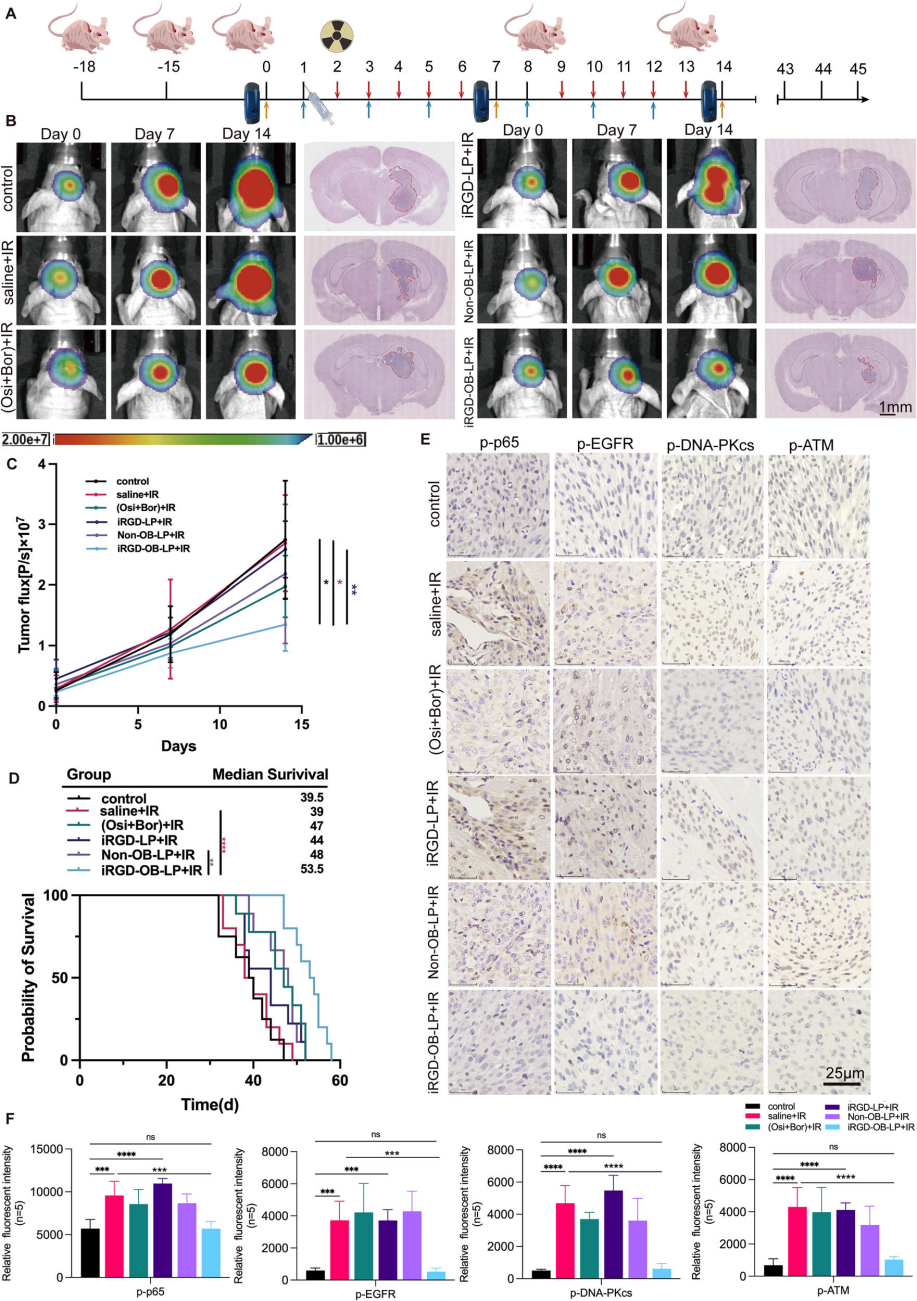
In vivo antitumor and radiotherapy sensitization effects of liposomes. **A** Schematic representation of the treatment regimen, illustrating the timeline of liposome administration (saline, iRGD-LP, non-OB-LP, or iRGD-OB-LP) and 2 Gy irradiation over the 14-day experimental period. Mice received treatments every 48 h, with daily irradiation for 5 days following the first liposome injection. Tumor sizes were assessed via intravital imaging on days 0, 7, and 14. **B** Representative in vivo tumor imaging on days 0, 7, and 14, showing tumor progression across treatment groups. Corresponding H&E staining of tumors on day 14 highlights tumor size differences among groups. Scale bar: 1 mm. **C** Statistical analysis of fluorescence intensity from tumor imaging. **D** Survival rates of GBM-bearing mice following different treatments. **E**, **F** Representative IHC staining images of tumor samples collected 2 h post-radiotherapy from GBM-bearing mice treated with various treatments, showing markers including p-p65, p-EGFR, p-DNA-PKcs, and p-ATM. Scale bar: 25 µm. ***p < 0.001, ****p < 0.0001.

To evaluate whether iRGD-OB-LP effectively improves radiotherapy sensitivity in radiotherapy-resistant GBM in vivo, an orthotopic glioblastoma model was generated through intracranial implantation of Diff-Luc cells into 4-week-old female BALB/c nude mice. Tumor growth was monitored for 15 days via in vivo bioluminescence imaging, after which mice were randomized into treatment groups receiving saline, an equivalent dose of the bortezomib-osimertinib (OB) combination, iRGD-LP, non-OB-LP, or iRGD-OB-LP via tail intravenous injection(60 µl per 10 g body weight every 48 h). Beginning one day post-initial treatment, fractionated irradiation (2 Gy per fraction) was administered daily for 5 consecutive days. Tumor progression was evaluated after each 5-day treatment cycle using bioluminescence imaging, with subsequent cycles conducted as needed over a 14-day period (Fig. 8A).

Longitudinal bioluminescence imaging on days 0, 7, and 15 revealed distinct therapeutic outcomes across groups. As shown in Fig. 8B, saline or iRGD-LP combined with irradiation failed to improve radiotherapeutic efficacy, exhibiting minimal tumor regression and no survival benefit. Non-OB-LP or free OB combined with irradiation induced moderate tumor suppression, whereas the iRGD-OB-LP + IR group demonstrated the most pronounced therapeutic response. Tumor growth curves confirmed significant suppression of tumor progression in this group compared to all others (Fig. 8B). Histopathological analysis via H&E staining further corroborated these findings, showing markedly reduced tumor burden in the iRGD-OB-LP + IR cohort (Fig. 8C). Survival analysis revealed a median survival of 53.5 days for the iRGD-OB-LP + IR group, significantly exceeding the 39.5 days observed in the saline + IR group (*p* < 0.0001) and outperforming the Non-OB-LP + IR group (*p* < 0.01) (Fig. 8D).

To investigate the mechanism of radiosensitization, tumor tissues harvested 2 h post-irradiation were subjected to immunohistochemical (IHC) analysis. iRGD-OB-LP treatment significantly suppressed activation of the EGFR and NF-κB pathways and their downstream signaling, with pronounced inhibition of phosphorylation in key DNA repair proteins ATM and DNA-PKcs compared to other treatment groups (Fig. 8E, F and Fig. S5C). Concurrently, we observed marked reductions in pAKT, Bcl-2, and IL-6 expression—biomarkers associated with apoptosis resistance and pro-tumorigenic inflammation (Fig. S5C). This dual impairment of DNA repair machinery combined with suppression of survival signaling pathways likely prolongs radiation-induced DNA damage persistence and attenuates cellular recovery mechanisms, thereby enhancing radiotherapy-induced cytotoxicity.

Collectively, these results demonstrate that iRGD-OB-LP potentiates radiotherapy in radioresistant GBM by targeting DNA repair mechanisms, thereby suppressing tumor growth and extending survival. This highlights its potential as a novel therapeutic strategy to overcome radioresistance in GBM.

## Discussion

Glioblastoma recurrence following radiotherapy remains a formidable clinical challenge, largely due to the survival and adaptive evolution of radioresistant GSCs within a heterogeneous tumor ecosystem. In this study, we established a novel glioblastoma model comprising radioresistant GSCs and differentiated (Diff) cells that recapitulate key clinical features of post-radiotherapy recurrence. While previous models predominantly focused on GSCs populations [39, 40], our dual-cell system uniquely captures the dynamic transition from therapy-resistant stem cells to differentiated progeny—a critical yet understudied aspect of tumor evolution during fractionated radiotherapy. This advancement addresses the clinical paradox where residual stem cells with acquired resistance often drive recurrence despite initial treatment response.

Through RNA sequencing analysis, we identified that the inhibition of EGFR and NF-κB signaling pathways represents a promising therapeutic strategy for overcoming radiotherapy resistance in glioblastoma. These findings align with prior studies [12, 13, 15, 16, 31], reinforcing the central role of these pathways in radioresistance. Building upon our previous work using siRNA to co-target RELA/p65 and EGFR [31], the current pharmacological approach employing bortezomib and osimertinib addresses key translational barriers. The transition from genetic to pharmacological intervention circumvents the technical challenges of nucleic acid delivery while leveraging clinically approved agents. Importantly, this strategy maintains dual inhibition of NF-κB and EGFR pathways, as evidenced by reduced p-p65 and p-EGFR levels, but introduces an additional dimension through ferroptosis induction—a mechanism absent in our prior siRNA-based system. A systematic review of *ClinicalTrials.gov* (July 2025; search parameters: Condition: Glioblastoma + Intervention: osimertinib; Condition: Glioblastoma + Intervention: bortezomib) reveals no clinical trials exploring either osimertinib combined with radiotherapy or co-delivery of osimertinib and bortezomib. While completed osimertinib trials (e.g., NCT03732352) involved Osimertinib for recurrent glioblastoma treatment, combined with 18F-FDG PET but not radiotherapy, and ongoing EGFR-targeted studies (NCT05256290) similarly exclude radiotherapy, bortezomib combinations remain limited to regimens such as NCT03643549 (active phase IB/II trial with temozolomide in recurrent glioblastoma), NCT00998010 (temozolomide/radiotherapy), and NCT01435395 (Bevacizumab/temozolomide)—all lacking EGFR co-inhibition. Our work, therefore, establishes the first platform for tumor-targeted co-encapsulation of osimertinib and bortezomib (iRGD-OB-LP) combined with radiotherapy.

Liposomes have long been investigated as efficient drug delivery systems for cancer therapy due to their ability to exploit the enhanced permeability and retention (EPR) effect, which facilitates passive accumulation in tumors via leaky vasculature [41, 42]. However, passive delivery alone often fails to achieve effective penetration into solid tumors due to stromal barriers and the dense extracellular matrix. To address this limitation, we functionalized our liposomes with iRGD, a tumor-penetrating peptide that actively facilitates transport across tumor vasculature and stroma. The iRGD modification confers three distinct advantages over conventional liposomes: enhanced tumor specificity through αvβ3/NRP1 targeting (Fig. 4A–E), improved intracellular delivery via increased lysosomal escape efficiency (Fig. 4E), and superior penetration through stromal barriers (Fig. 7B). However, the observed dose-dependent saturation of cellular uptake (Fig. S4B) suggests optimal dosing requires balancing receptor binding capacity with payload delivery—a consideration often overlooked in active targeting strategies.

Consistent with prior reports on integrin αVβ3-targeted glioma radiosensitization [43], we initially hypothesized that iRGD-modified liposomes (iRGD-LP) might exhibit intrinsic radiosensitizing properties. However, our data revealed that iRGD-LP alone failed to suppress irradiation-induced p65/EGFR activation or their downstream DNA repair effectors (ATM, DNA-PKcs), as evidenced by comparable γH2AX levels. While iRGD conjugation demonstrated mild anti-proliferative effects (Fig. S4C), its primary role appears limited to facilitating tumor-specific delivery rather than direct radiosensitization. This functional divergence from previous studies may stem from our moderate surface iRGD density, which prioritizes transporter-mediated transcytosis over sustained receptor inhibition. The potent radiosensitization observed with iRGD-OB-LP thus predominantly originates from Bortezomib/Osimertinib payloads, with iRGD acting as a precision delivery enhancer rather than a therapeutic agent per se.

Our mechanistic studies delineate a hierarchical model of radiosensitization in Diff cells. While iRGD modification enhanced liposomal uptake across both cell types, its therapeutic impact diverged significantly. Apoptosis emerged as the predominant cell death modality, particularly in radioresistant Diff cells (Fig. 5D, E and Fig. S4C), achieving comparable lethality to U87 cells despite the former’s higher intrinsic drug tolerance (Fig. 5A) and lower αvβ3/NRP1 expression (Fig. S3B). Radiation combination unveiled a secondary ferroptotic phenotype characterized by lipid droplet depletion (potentially via PPARγ and FABP7 inhibition) and ROS overaccumulation (Fig. S6B, E), though precise molecular mediators require further elucidation. This hierarchy suggests that while lipid metabolic alterations may create permissive conditions, targeted apoptosis induction through optimized NF-κB/EGFR co-inhibition represents the core therapeutic strategy against radioresistant GBM.

To address rapid liposome clearance by the reticuloendothelial system (RES), polyethylene glycol (PEG) was incorporated to extend circulation time [44, 45]. While iRGD-OB-LP exhibited significant tumor accumulation, residual hepatic/splenic deposition (Fig. 7B) highlights the need for further PEG optimization. This persistent off-target accumulation underscores inherent limitations of conventional liposomes, prompting consideration of alternative platforms like extracellular vesicles (EVs), which exhibit superior biological barrier penetration and biocompatibility [46]. Furthermore, while our models provide valuable insights, their inability to fully recapitulate human glioblastoma’s immunosuppressive microenvironment and reliance on specific cell lines necessitate cautious clinical extrapolation. Conclusions require stringent validation across complementary glioblastoma models to ensure broader applicability. Future investigations integrating immune checkpoint modulators or angiogenic regulators could strategically address these limitations, potentially synergizing with established radiosensitization mechanisms through multimodal therapeutic targeting.

## Conclusion

**Fig. 9:**
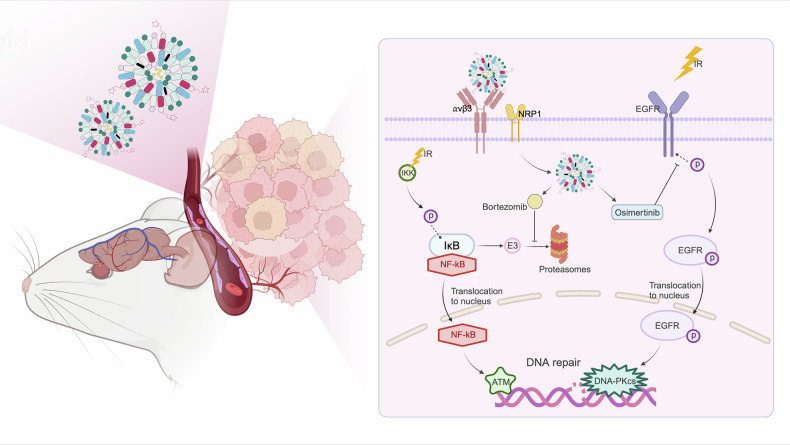
Schematic diagram of enhanced radiosensitivity in GBM using iRGD-OB-LP. The iRGD-OB-LP target and penetrate glioblastoma tumors, where they release Osimertinib to inhibit EGFR signaling and Bortezomib to suppress NF-κB activity. This dual pathway blockade attenuates DNA repair capacity, thereby synergizing with irradiation (lightning bolt) to amplify DNA damage and induce cell death. Created with Biorender.

This study highlights the potential of dual-functional brain-targeting liposomes with iRGD modification for the co-delivery of osimertinib and bortezomib to combat radioresistant glioblastoma (Fig. 9). Three critical advances emerge from our findings: First, the dual targeting of EGFR and NF-κB pathways induces synthetic lethality by simultaneously blocking radiation-induced survival signaling and DNA repair activation. Second, iRGD-mediated delivery achieves therapeutic-level drug accumulation in radioresistant tumor niches while minimizing systemic toxicity—a crucial advantage over conventional chemoradiation regimens. Third, the unexpected ferroptosis induction under combinatorial treatment expands the therapeutic landscape beyond apoptosis-focused strategies. While further optimization and preclinical evaluation are warranted, this multi-pronged approach provides a strong foundation for the development of advanced therapies targeting radioresistant GBM, with potential applicability to other therapy-resistant solid tumors.

## Supplementary information

Supplemental Material

Supplemental Material(western blot)

## Data Availability

RNA-seq data of this study is available in Gene Expression Omnibus (GEO) with accession code GSE285885.
