# Supplementary material for: GSCs differentiation model-informed nanotherapy: dual-functional brain-targeting liposomes with iRGD modification for co-delivery of osimertinib and bortezomib to combat radioresistant glioblastoma
Source: Cell Death Dis. 2025 Oct 21;16(1):738. doi: 10.1038/s41419-025-08083-0 (PMC12540775; doi:10.1038/s41419-025-08083-0)
Supplement: Supplementary file 1 — Supplemental Material [file 41419_2025_8083_MOESM1_ESM.docx]

**GSCs Differentiation Model-Informed Nanotherapy: Dual-functional Brain-targeting Liposomes with iRGD Modification for Co-delivery of Osimertinib and Bortezomib to Combat Radioresistant Glioblastoma**

Cuiying Xie^1,2#^, Jieyi Wu^1,2#^, Han Yang^3^, Ancheng Gu^1^, Wanwen Cao^1^, Xiangcao Yao^1^, Zhiyong Li^5^, Yuchun Niu^6^, Jianlong Li^4^*, Zhongyuan Xu^1^*, Bohong Cen^1^*

1. Clinical Pharmacy Center, Nanfang Hospital, Southern Medical University, Guangzhou 510515, Guangdong, China
2. School of Pharmaceutical Sciences, Southern Medical University, Guangzhou 510515, Guangdong, China
3. The Department of Plastic and Cosmetic Surgery, Nanfang Hospital, Southern Medical University, Guangzhou 510515, Guangdong, China
4. Department of Orthopedic Surgery, Nanfang Hospital, Southern Medical University, Guangzhou, 510515, Guangdong, China
5. Department of Neurosurgery, Nanfang Hospital, Southern Medical University, Guangzhou, 510515, Guangdong, China.
6. The First People's Hospital of Foshan, Cancer Hospital, Foshan, 528000, Guangdong, China.

# These authors contributed equally.

*Corresponding author

Email addresses: cenbohong22@i.smu.edu.cn (Bohong Cen), nflcyljd@smu.edu.cn (Zhongyuan Xu) and jianlongyx@163.com (Jianlong Li)

**Supplementary Material**

**
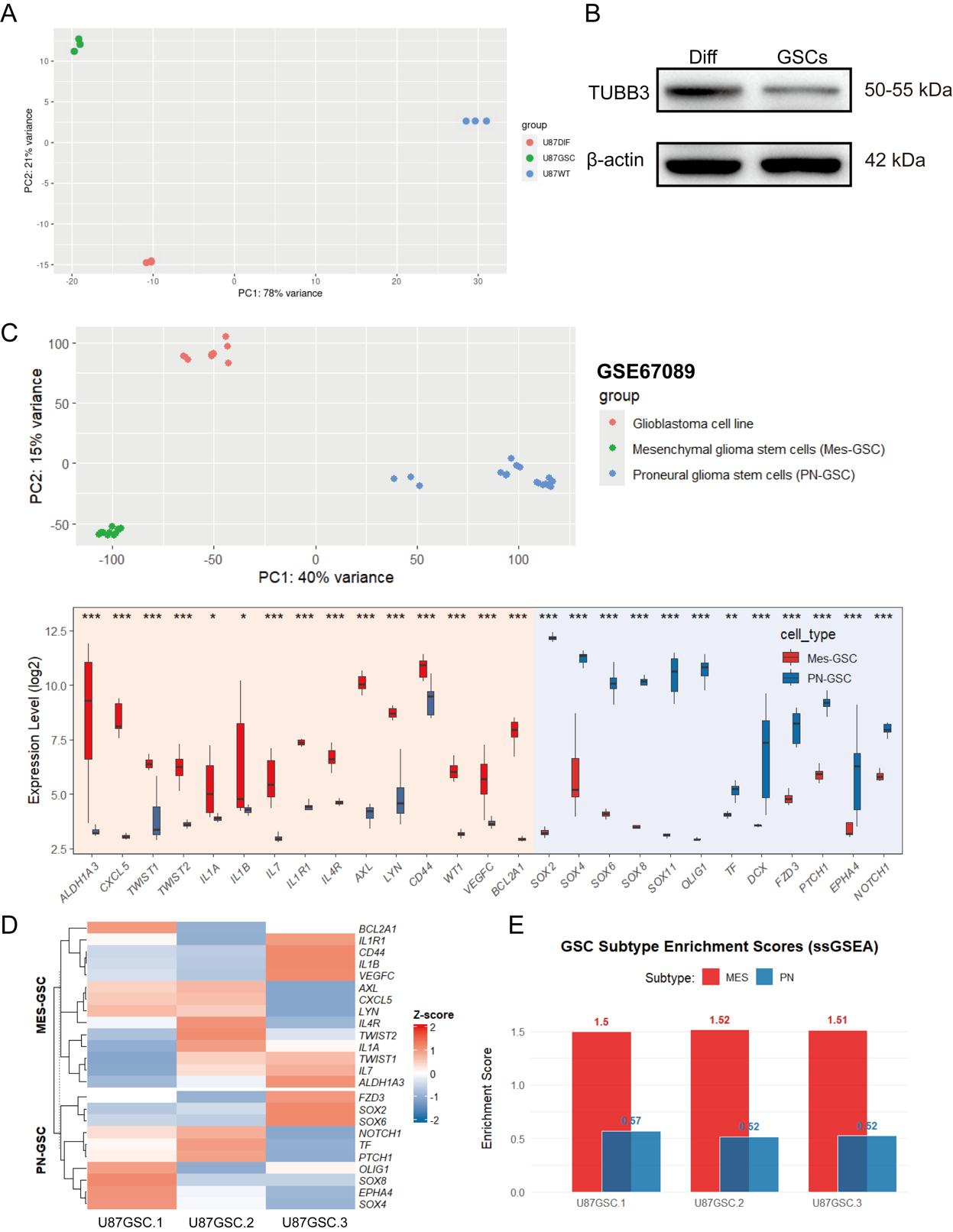
**

**Supplementary Figure 1. Molecular characterization of GSCs. (A)** PCA of RNA-Seq data comparing the three cell lines. **(B)** Western blot analysis of TUBB3 (neuronal marker) expression in GSCs and Diff cells. **(C)** Top: PCA of glioma samples from the GSE67089 dataset (GBM cell lines, MES-GSC, PN-GSC). Bottom: Box plots showing expression levels of mesenchymal (MES) and proneural (PN) signature genes in MES-GSCs and PN-GSCs from the GSE67089 dataset. **(D)** Heatmap displaying expression patterns of MES- and PN-associated signature genes in GSCs developed in this study (genes WT1, SOX11, and DCX were excluded due to zero Z-scores in all samples). **(E)** Single-sample gene set enrichment analysis (ssGSEA) scores using MES and PN signature genes, demonstrating elevated MES-like features in GSCs compared to PN traits.

**
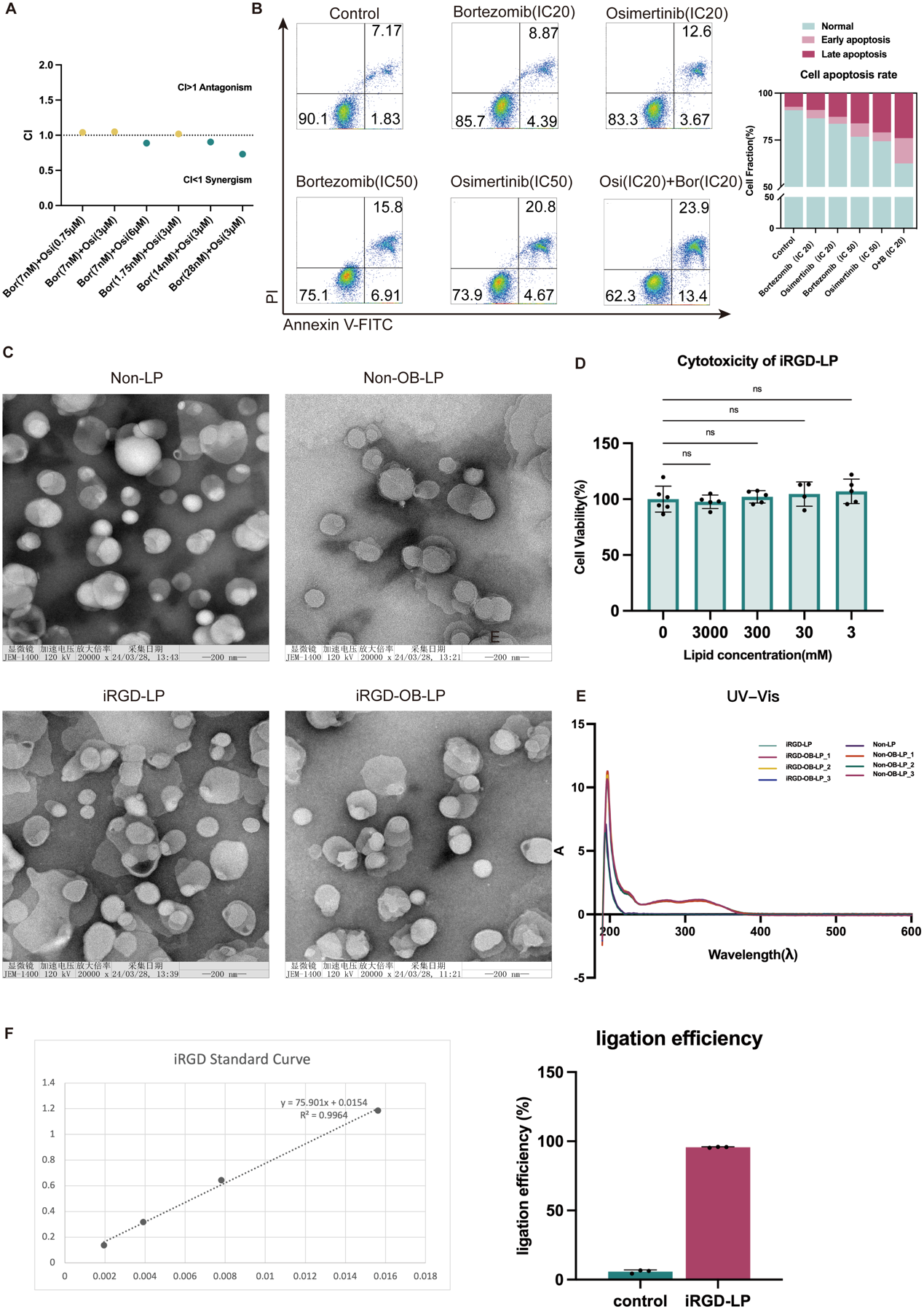
**

**Supplementary Figure 2. Feasibility of Two-Drug Combination and Liposome Characterization. (A)** CI analysis of the two-drug combination in Diff cells. CI > 1 indicates antagonism, CI = 1 suggests additivity, and CI < 1 reflects synergism. **(B)** Apoptosis analysis of Diff cells treated with Bortezomib (IC20 or IC50), Osimertinib (IC20 or IC50), or the combination of both drugs at IC20. **(C)** TEM images of different liposome formulations (Non-LP, Non-OB-LP, iRGD-LP, iRGD-OB-LP), scale bar = 200 nm. **(D)** Cell viability at different lipid concentrations. No significant inhibition of cell viability was observed at any of the tested concentrations. **(E)** UV-Vis absorption spectra of iRGD-OB-LP and Non-OB-LP, showing characteristic absorption peaks at 270 nm for Bortezomib and 330 nm for Osimertinib. **(F)**Standard curve of iRGD peptide and the ligation efficiency (%) of iRGD peptide in iRGD-LP.

**
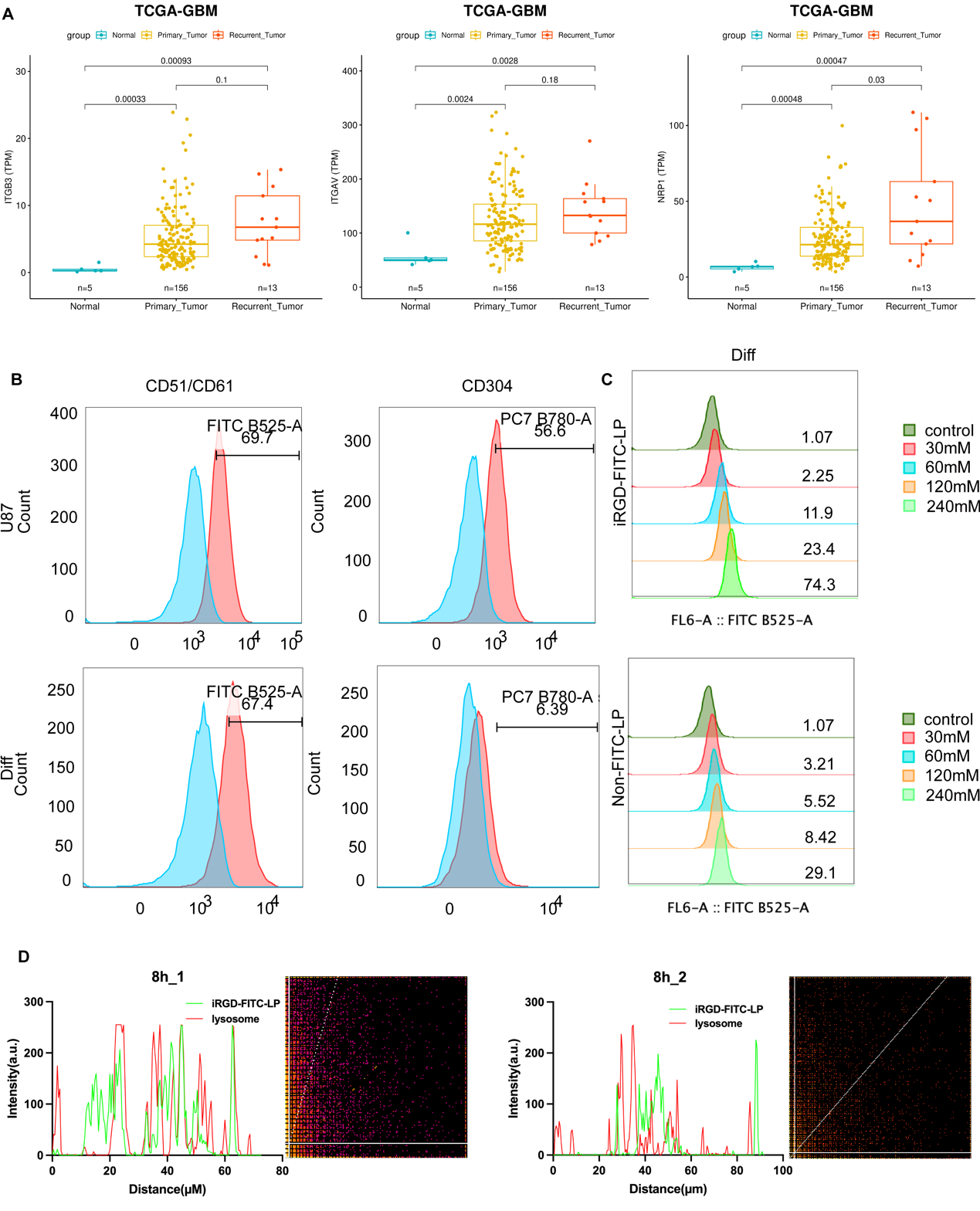
**

**Supplementary Figure 3. (A)** TCGA GBM data analysis illustrating the expression levels of αvβ3 integrin and NRP1 in both primary and recurrent GBM. **(B)** FCM result showing the surface expression of αvβ3 integrin and NRP1 in U87 and Diff cells. **(C)** FCM analysis demonstrating the uptake of iRGD-FITC-LP or Non-FITC-LP in Diff cells at different concentrations after 10 hours of treatment. **(D)** Co-localization analysis of iRGD-FITC-LP and lysosomes in Diff cells after 10 hours of uptake.

**
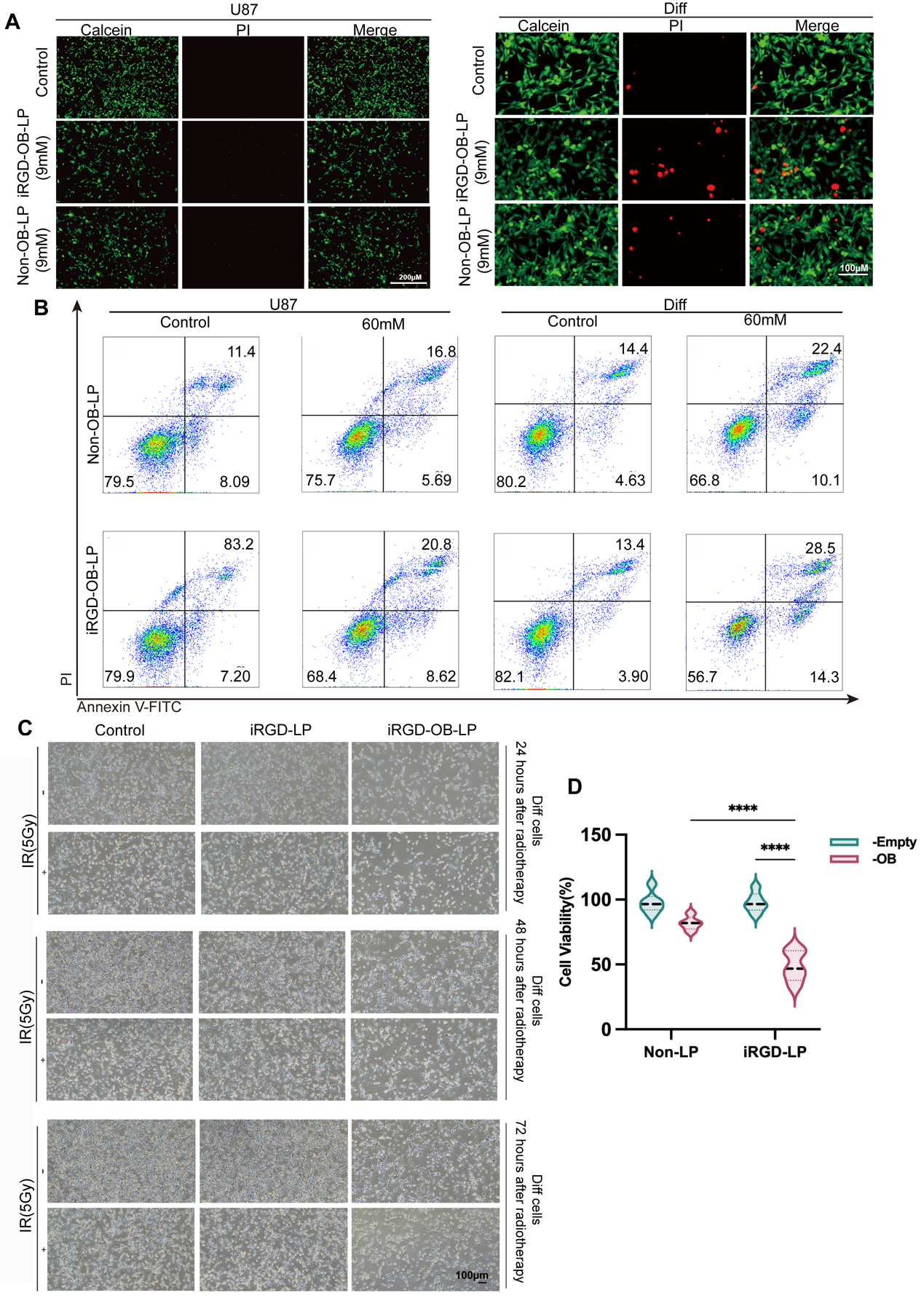
**

**Supplementary Figure 4. *In vitro* anti-tumor effects of liposomes. (A)** Dead-live staining of U87 and Diff cells after treatment with 9mM iRGD-OB-LP or Non-OB-LP for 10 hours, followed by 72 hours of normal culture. **(B)** Apoptosis analysis of U87 and Diff cells after treatment with 60mM iRGD-OB-LP or Non-OB-LP for 10 hours, followed by 72 hours of normal culture. **(C)** Representative images of Diff cells treated with 15 mM iRGD-LP or iRGD-OB-LP, with or without 5Gy irradiation. **(D)** Cell viability of Diff cells after treatment with drug-loaded liposomes or non-loaded liposomes for 10 hours, followed by 48 hours of normal culture, as measured by CCK8 assays.


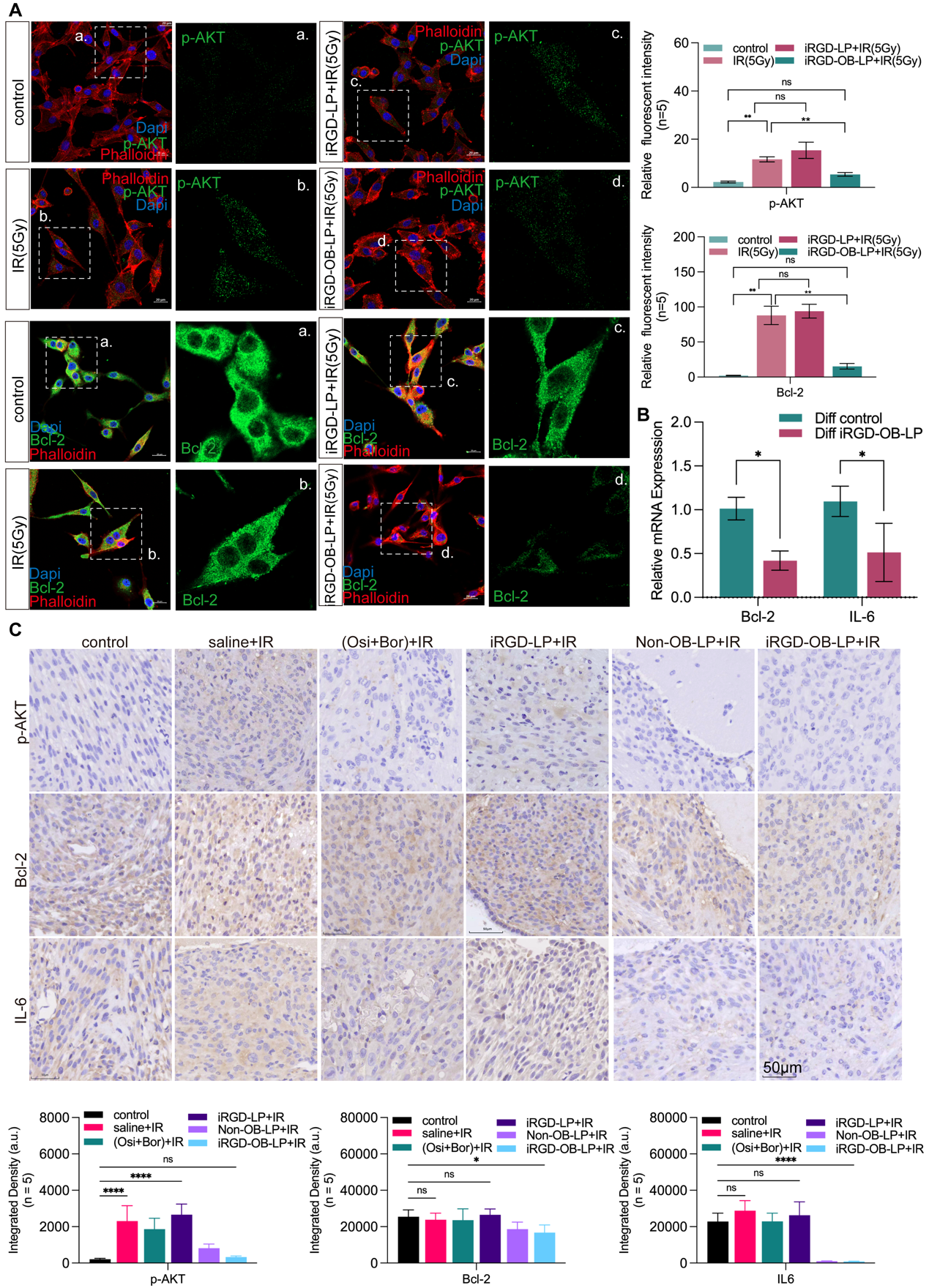


**Supplementary Figure 5. Supplementary experiments on liposomal mechanisms. (A)** Fluorescence images demonstrating that iRGD-OB-LP treatment significantly suppresses Bcl-2 expression and inhibits phosphorylation/activation of p-AKT at 1-2 h post-5 Gy irradiation in Diff cells. **p < 0.01. **(B)** Relative mRNA expression levels of Bcl-2 and IL-6 following iRGD-OB-LP treatment. **(C)** Representative IHC staining images of tumor samples collected 2 hours post-radiotherapy from GBM-bearing mice treated with various treatments, showing markers including p-AKT, Bcl-2 and IL-6. Scale bar: 50µm. *p < 0.05, ****p<0.0001

**
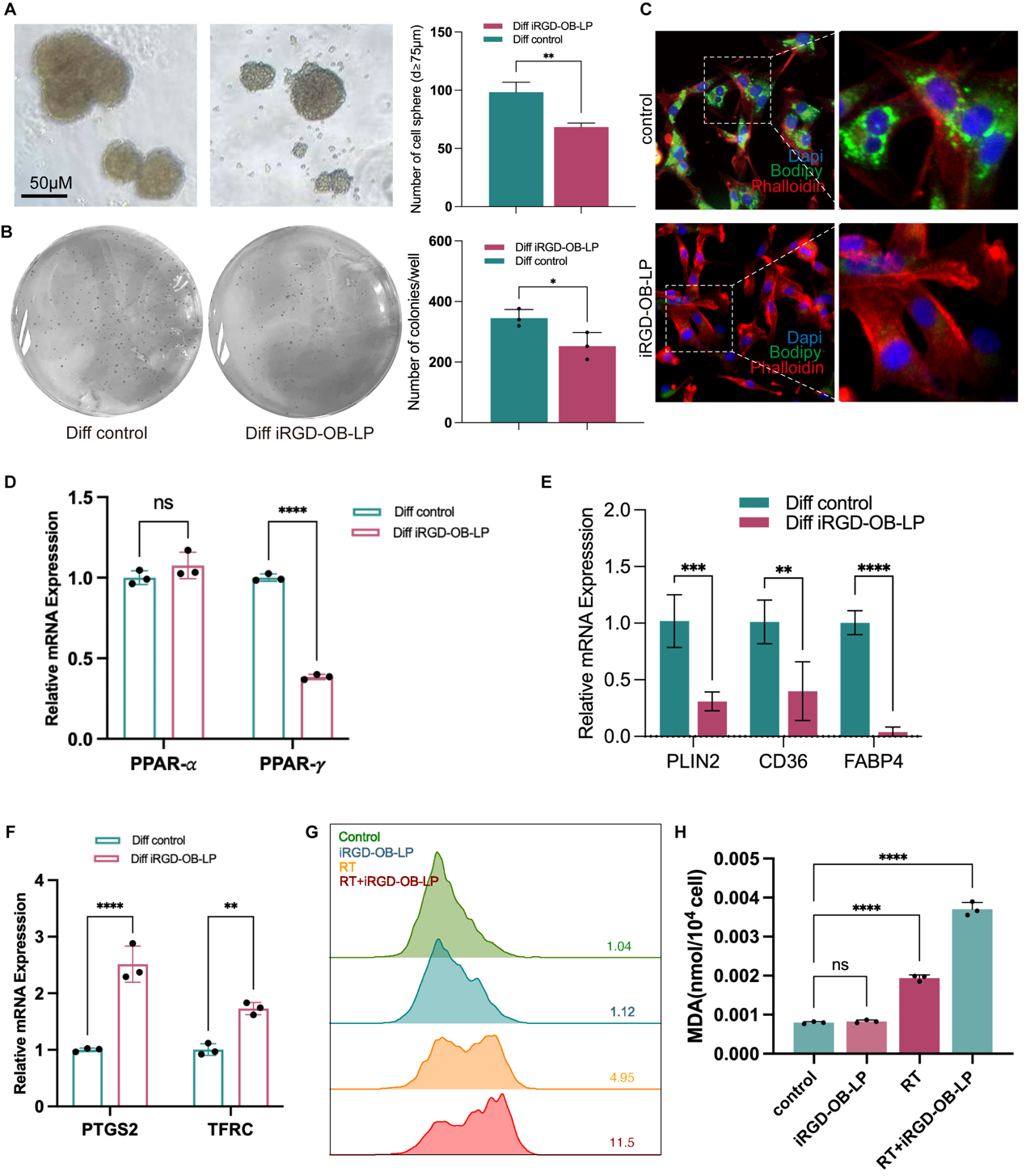
**

**Supplementary Figure 6. *In vitro* anti-tumor effects and mechanism of liposomes. (A)** Representative bright-field images and quantification of neurospheres formed by Diff cells under serum-free non-adherent conditions after 14 days. Treatments: control vs. iRGD-OB-LP. **p < 0.01. **(B)** Representative bright-field images and quantification of colony formation in Diff cells. Treatments: control vs. iRGD-OB-LP. *p<0.05. **(C)** Bodipy staining of Diff cells after treatment with 15 mM iRGD-OB-LP for 10 hours, followed by 72 hours of normal culture. **(D)** Relative mRNA expression levels of PPAR-𝛼 and PPAR-𝛾 in Diff cells after treatment with 15 mM iRGD-OB-LP for 10 hours, followed by 48 hours of normal culture. ****p<0.0001. **(E)** Relative mRNA expression levels of PLIN2, CD36 and FABP4 in Diff cells after treatment with 15 mM iRGD-OB-LP for 10 hours, followed by 48 hours of normal culture. **p<0.01, ***p<0.001,****p<0.0001. **(F)** Relative mRNA expression levels of PTGS2 and TFRC in Diff cells after treatment with 15 mM iRGD-OB-LP for 10 hours, followed by 48 hours of normal culture. **p<0.01, ****p<0.0001. **(G)** FCM analysis of ROS production in Diff cells 1 hour after 5Gy irradiation. **(H)** Malondialdehyde (MDA) assay in Diff cells post-5Gy irradiation, indicating lipid peroxidation levels. ****p<0.0001.

**
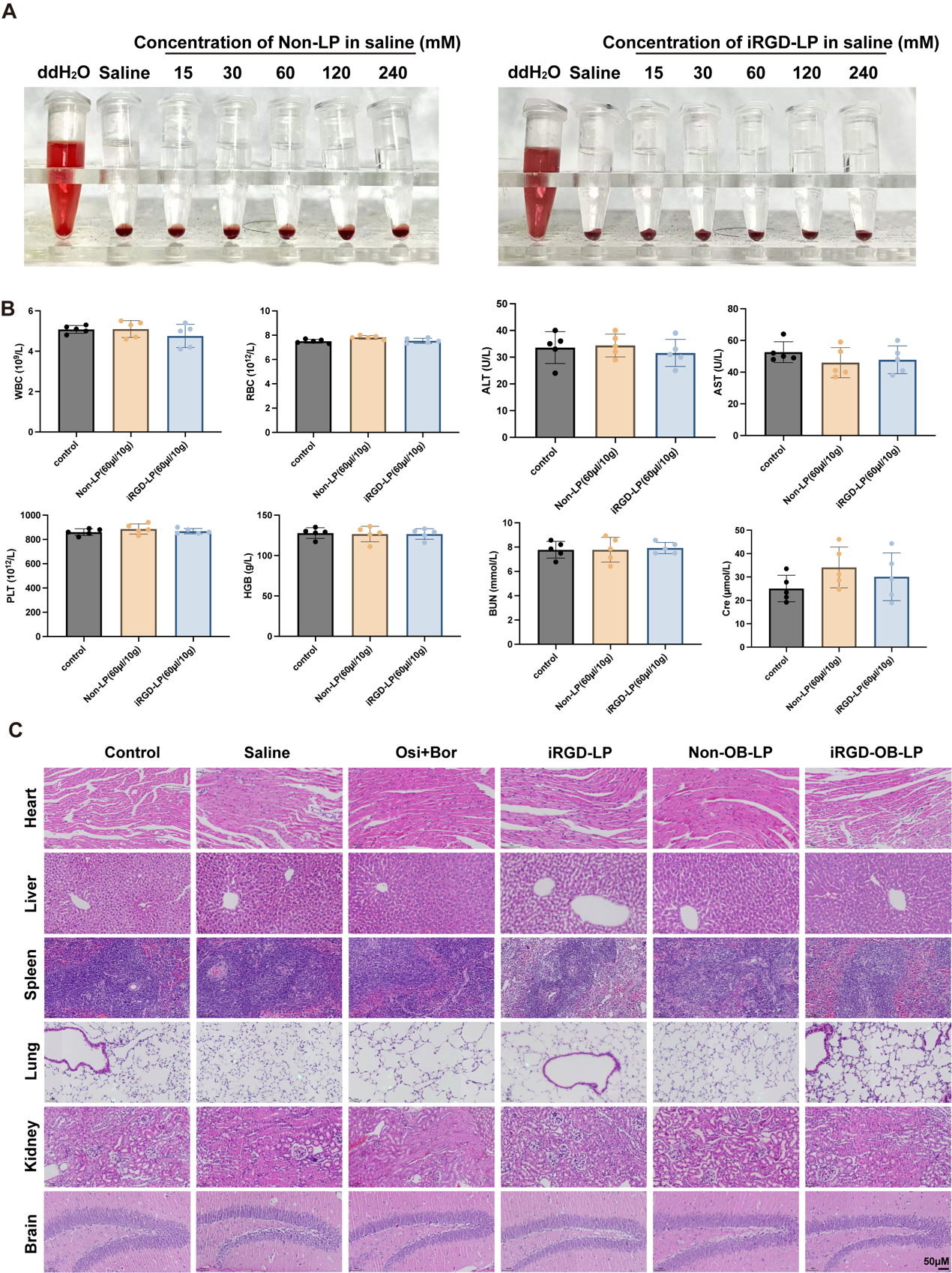
**

**Supplementary Figure 7. *In vivo* safety evaluation of liposomes. (A)** Hemolysis assay to assess the potential hemolytic effect of liposomes. **(B)** Blood routine and biochemical analysis in nude mice after administration of Non-LP or iRGD-LP. **(C)** H&E staining of major organs (heart, liver, spleen, lung, kidney, and brain) from nude mice following six rounds of treatment (Saline, Osimertinib combined with Bortezomib, iRGD-LP, Non-OB-LP, and iRGD-OB-LP).

**Antibody sources:**

Anti-CD133 ([proteintech], cat# [18470-1-AP)])

Anti-SOX2 ([proteintech], cat# [11064-1-AP])

Anti-FABP7 ([proteintech], cat# [51010-1-AP])

Anti-𝛾H2AX ([abcam], cat# [ab26350])

Anti-pp65 ([abcam], cat# [ab53489])

Anti-pEGFR ([abcam], cat# [ab316155])

Anti-pATM ([abcam], cat# [ab81292])

Anti-pDNAPKcs ([], cat# [ab124918])

Anti-Bcl2 ([proteintech], cat# [12789-1-AP])

Anti-pATK ([proteintech], cat# [66444-1-Ig])

Anti-IL6 ([proteintech], cat# [21865-1-AP])

Anti-Nestin ([proteintech], cat# [19483-1-AP]).

**Statistical analysis**

Statistical analyses used GraphPad Prism v10. Data are expressed as mean ± SD of ≥3 biological replicates (individual data points shown in figures; n specified in legends). Normality and variance homogeneity were verified for all datasets. Comparisons:

Two groups: Unpaired two-tailed t-test

Multiple groups: One-way ANOVA with Bonferroni post-hoc

Significance: *P<0.05, **P<0.01, ***P<0.001, ****p<0.0001.

Limitations: No blinding, randomization, or formal sample size calculation was used; sample sizes were empirically determined based on prior experimental variability.
