# Supplementary figures and images for: GSCs differentiation model-informed nanotherapy: dual-functional brain-targeting liposomes with iRGD modification for co-delivery of osimertinib and bortezomib to combat radioresistant glioblastoma
Source: Cell Death Dis. 2025 Oct 21;16(1):738. doi: 10.1038/s41419-025-08083-0 (PMC12540775; doi:10.1038/s41419-025-08083-0)

| 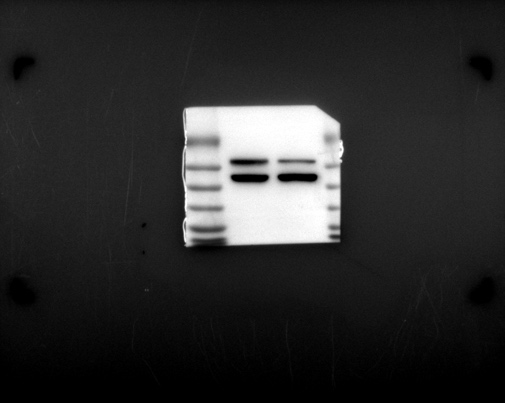 | | |
| --- | --- | --- |
| 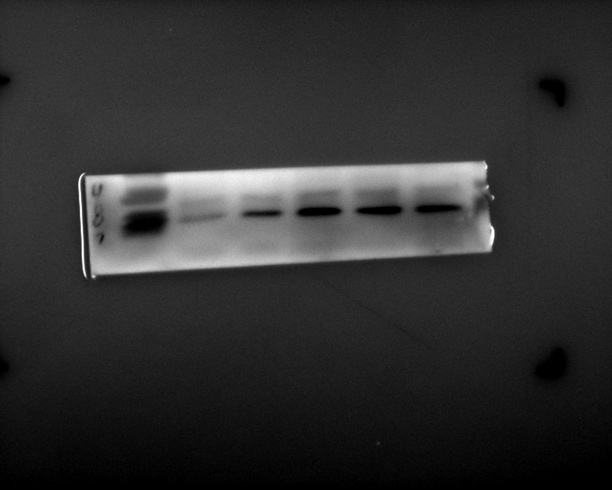 | 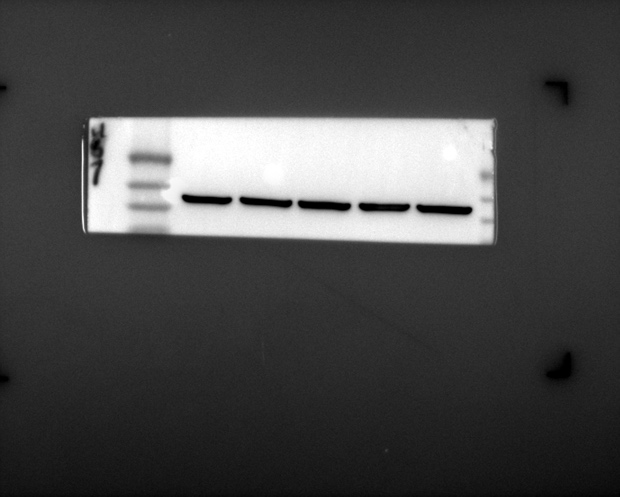 | |
| 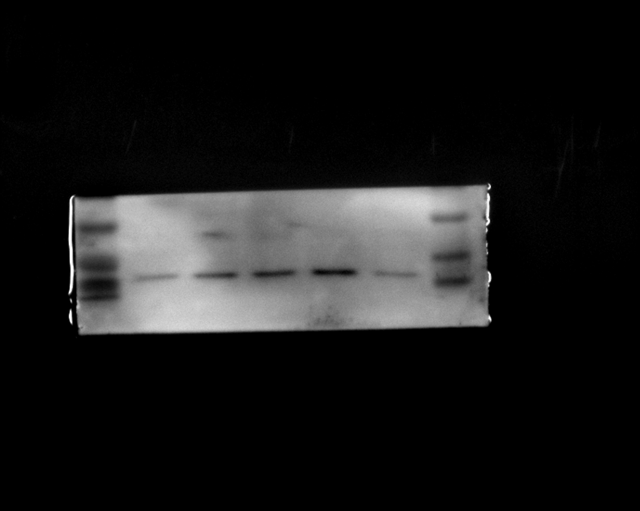 | | 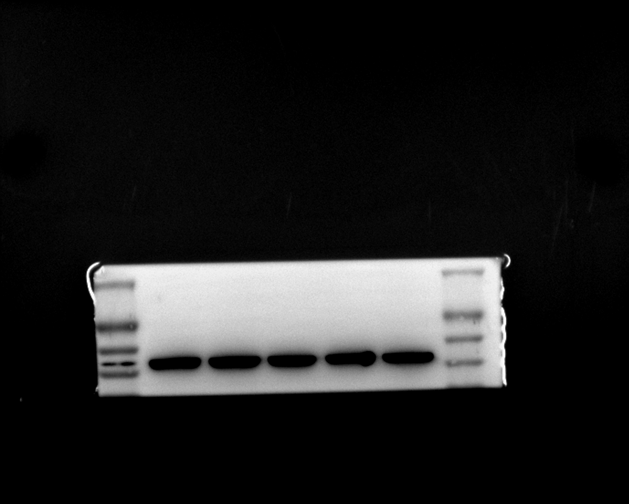 |
| 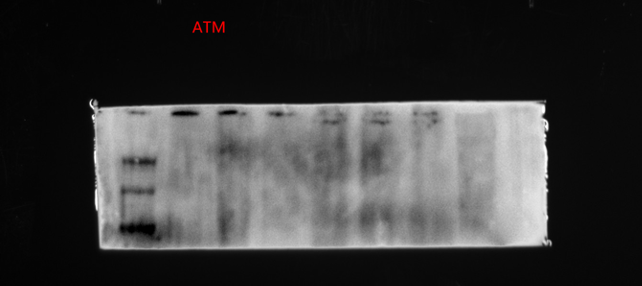 | | |
| 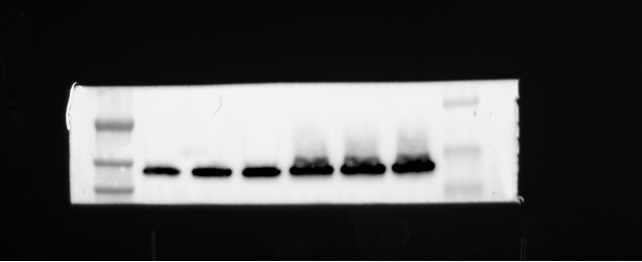 | | |
| 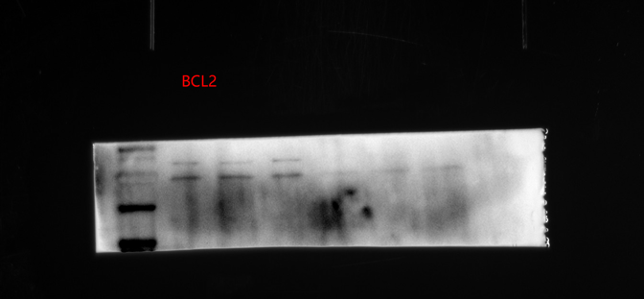 | | |
| 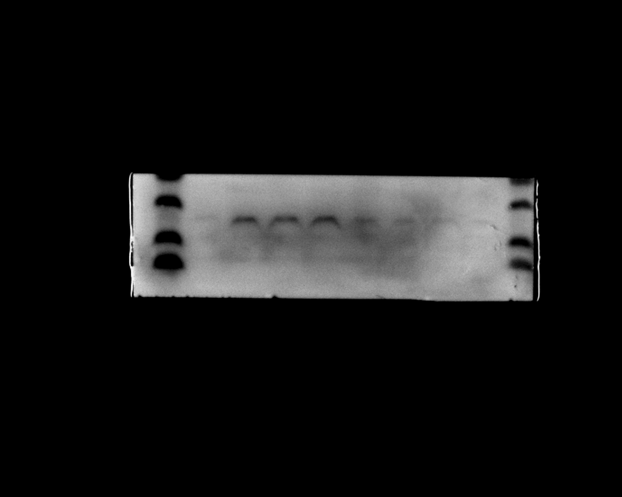 | 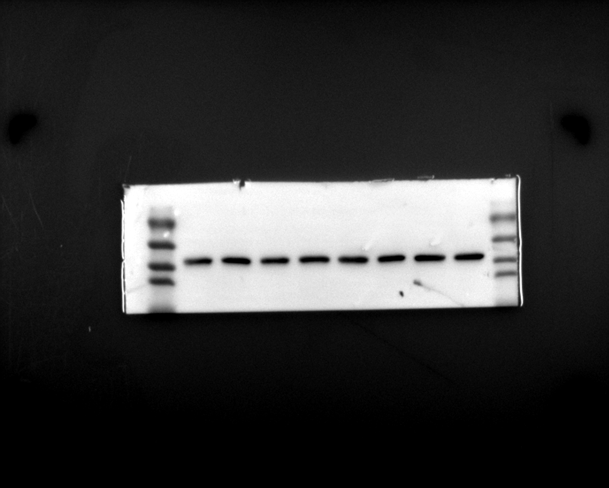 | |
| 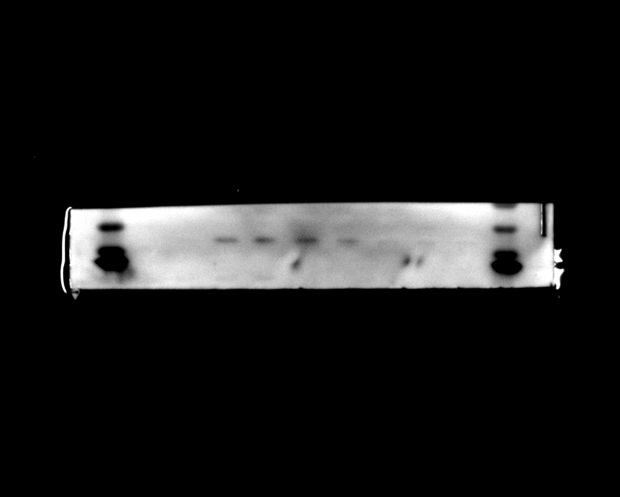 | 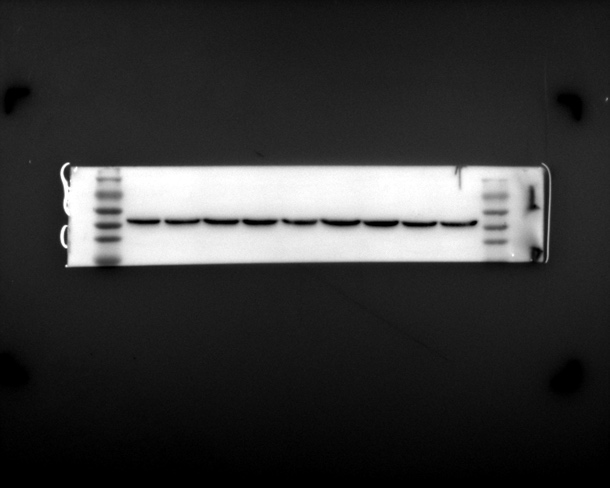 | |
| 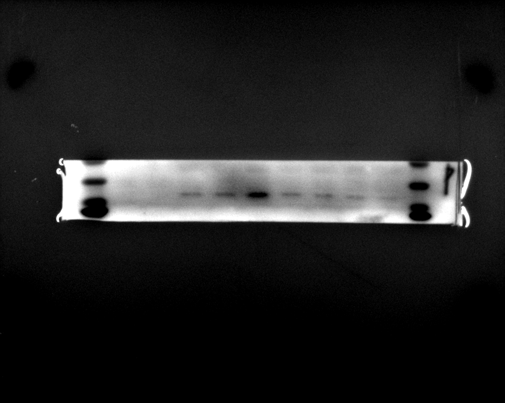 | 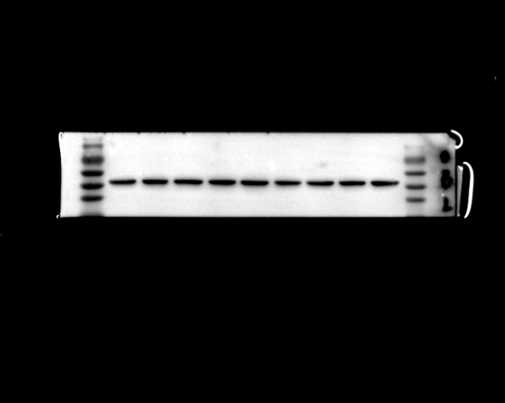 | |

Supplement: Supplementary file 2 — Supplemental Material(western blot) [file 41419_2025_8083_MOESM2_ESM.docx]
